# Structure and dynamics of a four-protofilament microtubule from Heimdallarchaeales α/β-tubulin

**DOI:** 10.1126/sciadv.aeh4305

**Published:** 2026-07-15

**Authors:** Linh T. Tran, Samson Ali, Tomoharu Matsumoto, Yosuke Yamazaki, Akihiro Narita, Makito Miyazaki, Robert C. Robinson

**Affiliations:** ^1^ RIKEN Center for Integrative Medical Sciences, 1-7-22 Suehiro-cho, Tsurumi-ku, Yokohama, Kanagawa 230-0045, Japan.; ^2^ RIKEN Center for Biosystems Dynamics Research, 2-2-3 Minatojima-minamimachi, Chuo-ku, Kobe, Hyogo 650-0047, Japan.; ^3^ Research Institute for Interdisciplinary Science, Okayama University, Okayama 700-8530, Japan.; ^4^ Department of Biological Science, Graduate School of Science, Nagoya University, Nagoya, Aichi 464-8601, Japan.; ^5^ Graduate School of Medicine, Science and Technology, Shinshu University, 3-1-1 Asahi, Matsumoto, Nagano 390-8621, Japan.; ^6^ School of Biomolecular Science and Engineering (BSE), Vidyasirimedhi Institute of Science and Technology (VISTEC), Rayong 21210, Thailand.

## Abstract

Eukaryotic microtubules are typically 13-protofilament tubes assembled from α/β-tubulin heterodimers that combine mechanical rigidity with dynamic instability. Homologous tubulins have been identified in Asgard archaea, the closest prokaryotic relatives to eukaryotes. Here, we characterize a heterodimeric α/β-tubulin system from Heimdallarchaeales. Biochemical reconstitution shows that *Heim–*α/β-tubulin forms a heterodimer that undergoes guanosine 5′-triphosphate–dependent polymerization with coupled nucleotide hydrolysis. Cryo–electron microscopy reveals that the polymers are composed of four-protofilament tubules, with microtubule-like lattices formed by conserved longitudinal interfaces and ball-and-socket lateral contacts. Single-filament imaging demonstrates intrinsic kinetic polarity and dynamic instability, while liposome encapsulation shows that microtubule growth generates forces sufficient to deform membranes. Despite their reduced protofilament number, *Heim*–α/β-microtubules share key structural and dynamic features with eukaryotic microtubules but exhibit lower bending stiffness and polymerization force. Thus, microtubule-like polymers can form from a range of protofilament numbers, with reduced architectures potentially adapted to small cellular dimensions and lower mechanical loads. Together, our results indicate expansion in microtubule protofilament number during eukaryogenesis.

## Teaser

A minimal four-protofilament microtubule reproduces key features of eukaryotic dynamics.

## INTRODUCTION

Microtubules are dynamic cytoskeletal polymers that play central roles in eukaryotic cell morphology, division, motility, and intracellular transport (*1*–*4*). They are assembled from obligate heterodimers of α- and β-tubulin, which polymerize into longitudinal protofilaments that associate laterally to form a hollow cylindrical tube, typically composed of 13 protofilaments. This architecture confers high bending rigidity compared to actin filaments, enabling microtubules to span large cellular distances and resist compressive forces. In contrast to actin filaments, microtubules exhibit dynamic instability, characterized by stochastic transitions between phases of growth and rapid depolymerization (catastrophe) (*5*, *6*). These dynamics arise from guanosine 5′-triphosphate (GTP) binding and hydrolysis within the β-tubulin subunit. Incorporation of GTP-bound tubulin at the fast-growing plus end stabilizes the lattice, whereas subsequent GTP hydrolysis weakens lateral interactions between protofilaments. Loss of the stabilizing GTP cap promotes protofilament curvature, lattice destabilization, and rapid depolymerization. These intrinsic structural and dynamic properties are harnessed during mitosis and meiosis to assemble the mitotic spindle and drive chromosome segregation, and in motile structures such as cilia and flagella, where microtubule rigidity supports force generation. In addition, motor proteins such as kinesin and dynein exploit microtubule tracks for directed intracellular transport (*4*). The behavior of microtubules is further tuned by a diverse set of microtubule-associated proteins that regulate their dynamics, organization, and mechanical properties in a context-dependent manner (*6*).

Beyond their near-universal presence in eukaryotes, tubulin homologs have also been identified in diverse prokaryotic lineages. In bacteria, members of the genus *Prosthecobacter* (phylum Verrucomicrobiota) encode heterodimeric BtubA/B tubulins that assemble into microtubule-like structures. In vitro, BtubA/B forms dynamically unstable four-protofilament tubules (*7*), whereas in vivo filaments with five protofilaments have been observed (*8*). These bacteria are typically 1 to 2 μm in size (*9*). Within archaea, tubulin homologs are found in members of the Promethearchaeati (Asgard) superphylum, the closest known prokaryotic relatives of eukaryotes (*10*). Odinarchaeota encode a single tubulin (OdinTubulin) that assembles into tubules where the protofilaments encircle the diameter of the tubule (*11*), in which all subunits retain catalytic activity, consistent with homopolymeric assembly; however, these organisms have not yet been isolated. In contrast, *Candidatus* Lokiarchaeum ossiferum encodes α/β-like tubulin homologs that form heterodimers and assemble into five-protofilament microtubules (*12*). Lokiarchaeal cells are typically 1 to 1.5 μm in diameter but can extend long protrusions up to ∼15 μm (*13*). These dimensions compare with current estimates for the last eukaryotic common ancestor (LECA), which is thought to have been a relatively small (<25 μm), unicellular, and likely flagellated organism (*14*). These observations highlight the structural and functional diversity of prokaryotic tubulin systems and raise questions about how tubulin architecture adapts to different cellular contexts. To address this, we identified and characterized a previously unrecognized tubulin system in Heimdallarchaeales.

## RESULTS

### Heimdallarchaeales tubulin sequences and phylogeny

**Fig. 1.**
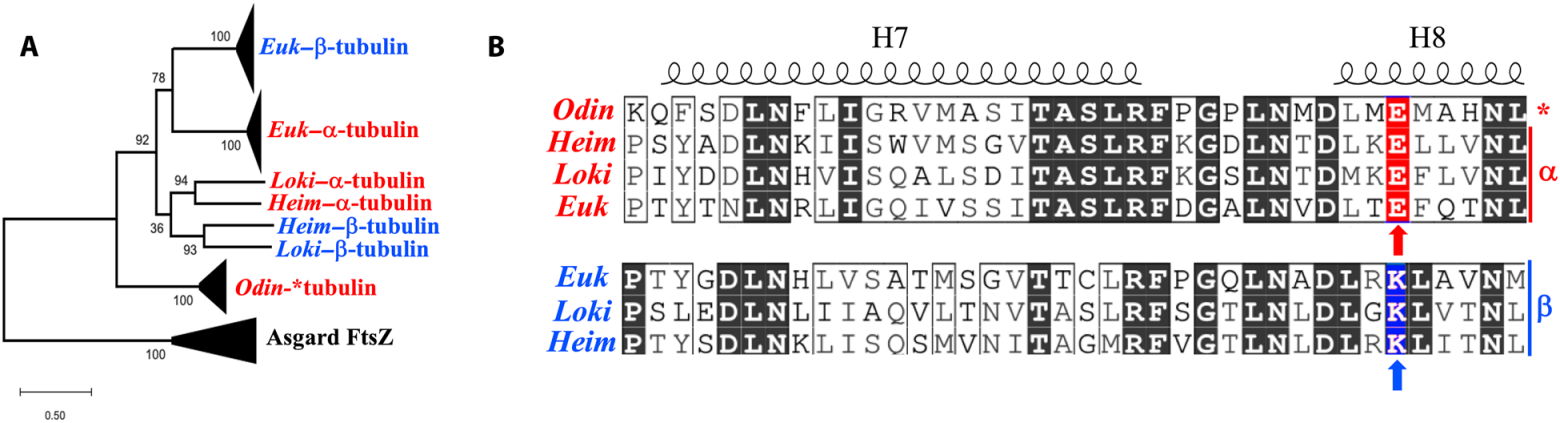
Phylogenetic relationships of Asgard tubulins. (**A**) Maximum likelihood phylogenetic tree of selected polymerizing eukaryotic (*Euk*) and Asgard tubulins and Asgard FtsZ sequences as an outgroup. Red and blue indicate tubulins with a catalytic glutamate or noncatalytic lysine residue, respectively. Asgard FtsZ sequences, which are structurally homologous to tubulins, are used to root the tree. (**B**) Sequence alignment of the nucleotide-sensor motif (tubulin helices H7 and H8), which connects the two nucleotide-binding sites within tubulin protofilaments (*11*). Red and blue arrows indicate the glutamate or lysine residue, respectively.

We identified an operonic pair of tubulin homologs in a 4.4-Mb metagenome-assembled genome (MAG) of a *Candidatus Heimdallarchaeota* archaeon (*15*) [National Center for Biotechnology Information (NCBI) ASM2988233v1] by BLAST searches using OdinTubulin (*11*) as the query. The reported MAG was obtained from estuarine surface sediment collected near San Francisco, USA (NCBI SAMN30443096). The two genes are adjacent and encoded in the same orientation, consistent with coexpression and potential functional coupling. Multiple structure-guided sequence alignment with representative polymerizing Asgard archaea and eukaryotic tubulins, followed by maximum likelihood phylogenetic analysis, revealed that the two distinct Heimdallarchaeales (*Heim*) tubulins branch separately with corresponding Lokiarchaeota/Promethearchaeaceae (*Loki*) homologs (*16*), forming two sister clades (Fig. 1A and fig. S1). These *Loki*/*Heim* pairs are distinct from, but closely related to, the eukaryotic α- and β-tubulin clades, which themselves resolve into two sister groups. OdinTubulins form a separate, more basal clade relative to *Heim*, *Loki*, and eukaryotic tubulins, whereas the structurally homologous Asgard FtsZ proteins branch more deeply and serve as an outgroup to all tubulin homologs.

Inspection of conserved catalytic elements further supports functional divergence within the *Loki*/*Heim* pairs. In canonical eukaryotic tubulin protofilaments, α-tubulin contributes a catalytic glutamate to the longitudinal interface, whereas β-tubulin has a lysine substitution at this position, which abolishes catalytic activity and stabilizes the GTP-bound state within the obligate α/β-tubulin heterodimer. Examination of the nucleotide-sensor motif (tubulin helices H7 and H8) (*11*), which couples adjacent nucleotide-binding sites within tubulin protofilaments (Fig. 1B), revealed a similar partitioning in *Loki* and *Heim* tubulins. One branch retains the catalytic glutamate, whereas the other branch encodes a lysine residue at the equivalent position. This complementary distribution predicts formation of a stable α/β-dimer, analogous to the *Loki* and eukaryotic tubulin pairs. In contrast, OdinTubulin is encoded as a single gene and lacks a corresponding lysine-bearing partner, consistent with its homopolymeric assembly (*11*). Given its phylogenetic position basal to the glutamate/lysine-diverged clades, we refer to OdinTubulin here as a proto-tubulin (*Odin*-*tubulin) and designate the *Loki*, *Heim*, and eukaryotic paralogs as α-like (Glu-bearing) and β-like (Lys-bearing) tubulins: *Loki*–α-tubulin, *Heim*–α-tubulin, and eukaryotic α-tubulin and *Loki–*β-tubulin, *Heim–*β-tubulin, and eukaryotic β-tubulin, respectively.

Tubulins are rare within Asgard genomes (*10*), and α- and β-like sequences from *Loki* and *Heim* form a distinct clade from their eukaryotic counterparts (Fig. 1A). The node separating *Loki*/*Heim–*α/β-tubulins from eukaryotic α/β-tubulins is strongly supported (92%). This topology does not suggest direct (α to α and β to β) ancestry of eukaryotic α- and β-tubulins from their respective Asgard homologs. Given the patchy distribution of tubulins across Asgard lineages and the separation of eukaryotic and Asgard α/β clades, these data are consistent with scenarios involving horizontal gene transfer. One possibility is that an *Odin* ancestor acquired a single tubulin gene from a proto-eukaryotic or related lineage, whereas paired α/β-like tubulins in *Loki* and *Heim* were transferred as a prediverged heterodimeric module from a lineage distinct from the one giving rise to modern eukaryotic α/β-tubulins. However, vertical inheritance followed by extensive differential gene loss across Asgard clades cannot be entirely excluded on the basis of the currently available genomic data (*10*). In either scenario, the sporadic distribution of tubulins among Asgard archaea would imply nonessential or lineage-specific roles, in contrast to the essential and conserved roles of tubulins in eukaryotes.

### Biochemical characterization of *Heim–*α/β-tubulin

**Fig. 2.**
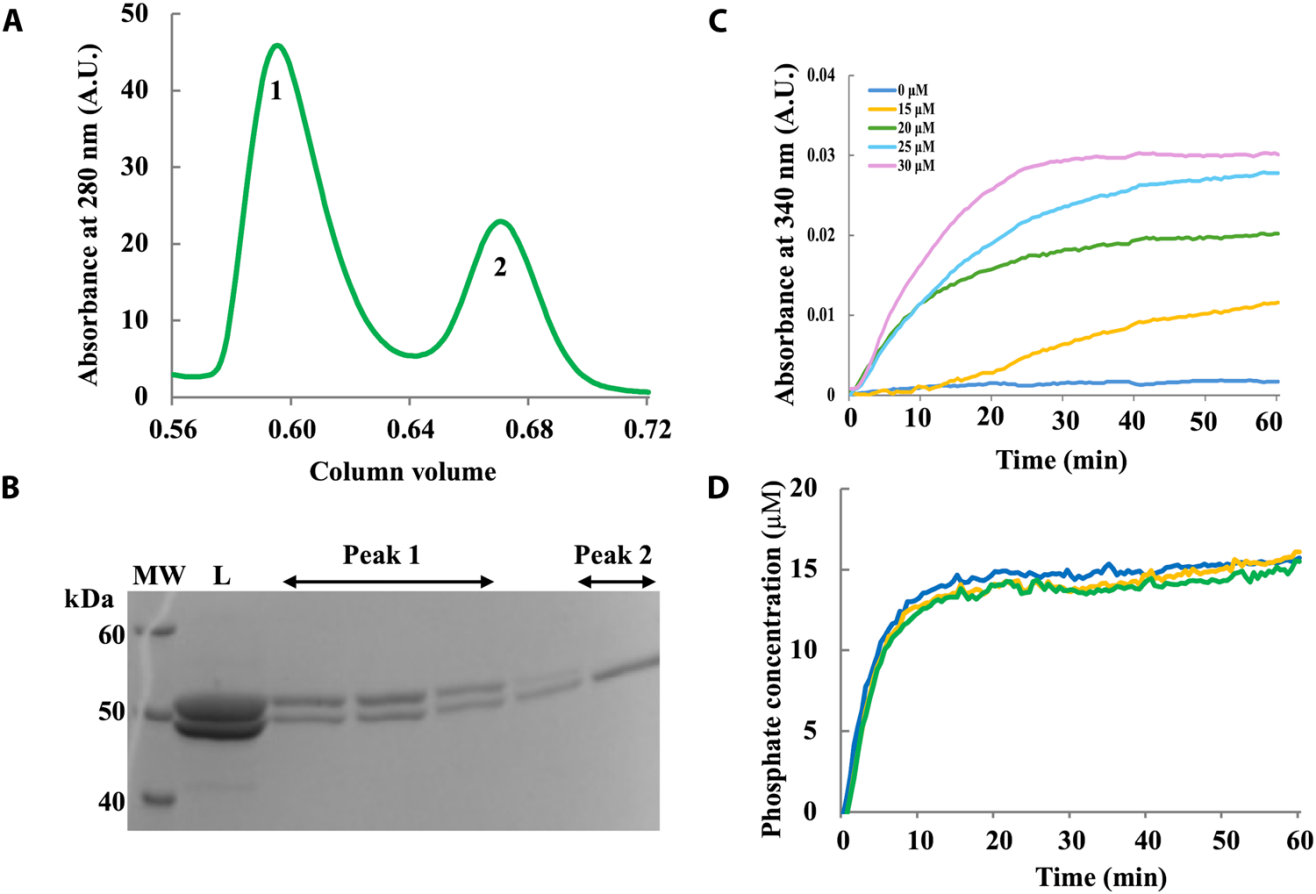
Biochemical characterization of *Heim–*α/β-tubulin. (**A**) Size exclusion chromatography profile and (**B**) SDS-PAGE image of *Heim*–α/β-tubulin. *Heim*–α/β-tubulin heterodimer complex (peak 1) eluted before *Heim*–α-tubulin (peak 2). A.U., arbitrary units; L, load sample; MW, molecular weight. (**C**) Polymerization time course of *Heim*–α/β-tubulin (0 to 30 μM) in the presence of 2 mM GTP at 37°C. The polymerization activity was monitored by measuring absorbance at 340 nm. (**D**) GTP hydrolysis activity of *Heim*–α/β-tubulin (15 μM) in the presence of 1 mM GTP at 37°C. Pi release was monitored using the EnzChek detection reagent by measuring absorbance at 360 nm. Three independent experiments were performed. Further SDS-PAGE, gel filtration, depolymerization, and hydrolysis activity data are provided in figs. S2 and S3.

To determine whether *Heim*–α-tubulin and *Heim–*β-tubulin form a stable heterodimer and support nucleotide-dependent polymerization, we expressed the proteins either individually or by coexpression in *Escherichia coli*. *Heim*–α-tubulin, when expressed alone, could be purified (fig. S2A). In contrast, expression of *Heim–*β-tubulin alone yielded low protein levels and insufficient material for biochemical characterization beyond gel filtration analysis (fig. S2B), suggesting reduced stability or solubility in the absence of its partner subunit. Coexpression of His-tagged *Heim*–α-tubulin with untagged *Heim–*β-tubulin yielded a soluble complex that coeluted as a large dimer peak and smaller monomer peak during size exclusion chromatography, consistent with stable heterodimer formation and residual *Heim*–α-tubulin monomer (Fig. 2, A and B, and fig. S2, A and C). Separation of the two subunits was achieved using SDS–polyacrylamide gel electrophoresis (PAGE), confirming the presence of both α- and β-like proteins in purified fractions (Fig. 2B and fig. S2, D and E).

We next examined whether purified *Heim*–α/β-tubulin undergoes assembly in vitro. Light scattering revealed a concentration-dependent increase in absorbance at 340 nm at 37°C in the presence of 2 mM GTP consistent with polymerization (Fig. 2C). Polymerization reached a plateau after ∼15 min at protein concentrations of 20 to 30 μM, indicating robust filament populations under these conditions. In contrast, replacement of GTP with 2 mM guanosine diphosphate (GDP) resulted in only minimal light scattering, even at the highest protein concentration tested (fig. S3A). These results indicate that *Heim*–α/β-tubulin assembly is GTP dependent and that the GDP-bound state is assembly incompetent or destabilized, analogous to the behavior of eukaryotic tubulin.

To quantify nucleotide turnover during assembly, we measured inorganic phosphate (Pi) release using a continuous enzymatic assay (*17*). At 15 μM *Heim*–α/β-tubulin, polymerization kinetics closely paralleled Pi accumulation, reaching ∼15 to 16 μM Pi after 60 min (Fig. 2D and fig. S3, B and C). This correspondence indicates approximately one GTP hydrolysis event per incorporated heterodimer. The observed ∼1:1 stoichiometry resembles that of eukaryotic tubulin, in which α-tubulin contains a nonexchangeable, nonhydrolyzing nucleotide-binding site (N-site), whereas β-tubulin harbors the exchangeable, GTP-hydrolyzing nucleotide-binding site (E-site) (*18*). Together, these data demonstrate that *Heim*–α/β-tubulin forms a stable heterodimer capable of GTP-dependent polymerization coupled with stoichiometric GTP hydrolysis.

### Structure of the *Heim–*α/β-tubulin polymers

Having established that *Heim*–α/β-tubulin forms a stable, GTP-dependent heterodimer capable of polymerization, we next examined the morphology of the resulting assemblies by electron microscopy of negatively stained samples. Incubation of *Heim*–α/β-tubulin at 10 to 20 μM in Pipes buffer (pH 6.8) containing 2 mM GTP at 37°C for 10 to 20 min produced abundant filaments (fig. S4, A to F). Twofold dilution of the samples immediately before grid application revealed distinct filament morphologies, including bundled, bent, and curved filaments (fig. S4, C and E), indicating structural plasticity reminiscent of tubulin polymers. Filament density increased with protein concentration, and at 30 μM, polymerization occurred rapidly, with extensive filament formation detectable after short incubation times (5 min; fig. S4F). Supplementation of the polymerization buffer with 10 mM potassium phosphate further increased filament abundance and promoted more uniform GTP-dependent assemblies at equivalent protein concentrations and incubation times (fig. S4, G to K). These conditions were therefore adopted for subsequent cryo–electron microscopy (cryo-EM) experiments. Consistent with light scattering measurements, replacement of GTP with GDP abolished filament formation, and only dispersed particles were observed (fig. S4H), confirming that assembly is strictly GTP dependent.

**Fig. 3.**
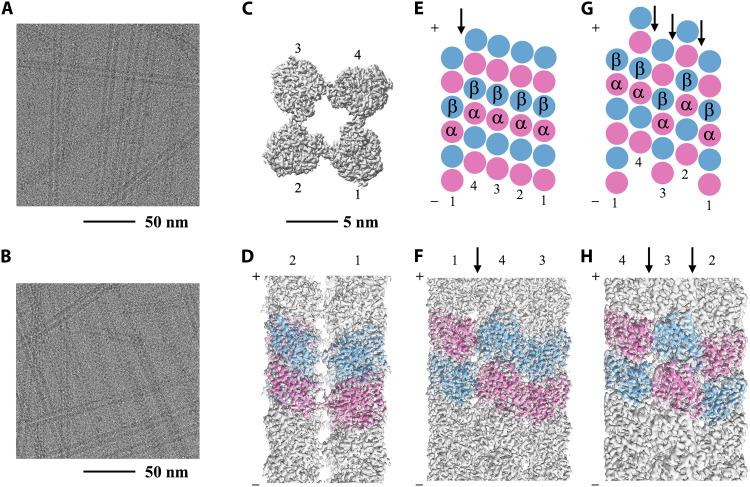
*Heim–*α/β-tubulin 4-protofilament microtubule. (**A** and **B**) Representative cryo-EM images of *Heim*–α/β-tubulin filaments. (**C**) Top and (**D**) side views of the cryo-EM map for the one-seam *Heim*–α/β-microtubule architecture. *Heim*–α-tubulin (pink) and *Heim–*β-tubulin (light blue) protomer structures are placed in the side view. (**E**) Cartoon depicting the one-seam contacts. The arrow indicates the seam and the protofilaments are numbered. (**F**) Side view of the one-seam architecture rotated by 45° to show the seam (arrow). (**G**) Cartoon depicting the three-seam contacts. (**H**) Side view of the three-seam architecture showing two seams (arrows). + and – indicate the plus and minus ends of the filaments, where the plus end is defined by the presence of the nucleotide-binding site.

To obtain high-resolution structural insight into *Heim*–α/β-tubulin filaments, we collected cryo-EM images, followed by three-dimensional (3D) reconstruction. The cryo-EM images reveal straight train track–like filaments, typical of projections of 3D tubules (Fig. 3, A and B, and fig. S5). Most filament ends were blunt, but a small proportion exhibited frayed conformations with short, curved filaments extending away from the tubule (fig. S6). The filaments assemble into four-protofilament tubules (Fig. 3, C and D), similar to the bacterial BtubA/B system (*7*). However, 3D classification revealed structural heterogeneity among the tubules (fig. S7). We therefore subdivided the filament segments into two major structural classes. The dominant class yielded a 2.8-Å reconstruction of the tubule (Fig. 3, C and D, and fig. S8) and a 2.4-Å reconstruction of the protofilament, obtained by averaging the four strands. In this one-seam architecture, lateral contacts are microtubule-like: α-tubulin interacts laterally with α-tubulin, and β-tubulin interacts laterally with β-tubulin throughout most of the lattice, except at a single seam where α-tubulin laterally associates with β-tubulin (Fig. 3, E and F). A second class yielded a 3.9-Å reconstruction corresponding to a three-seam architecture (fig. S8). In this arrangement, adjacent protofilaments interact primarily through heterologous α-β lateral contacts, resulting in three α-β seams and a single homologous α-α or β-β interface at the closure point (Fig. 3, G and H). In both structural classes, the left-handed tubule is formed by neighboring protofilaments that rise approximately by one-quarter of a protomer height and rotate by ∼90°, producing a four-subunit *Heim*–α/β-tubulin offset ring that spirals around the tubule. These structures reveal that the filaments observed by negative-stain electron microscopy correspond to closed tubules composed of four protofilaments.

The two architectures can be understood by considering the tubule as a four-protofilament sheet that rolls into a cylinder. A sheet composed of homologous lateral contacts closes to form a tubule containing a single heterologous α-β seam, analogous to that observed in eukaryotic microtubules. In contrast, a sheet composed of alternating α-β contacts closes, such that three of the four lateral interfaces form α-β seams, while the remaining interface forms a homologous α-α or β-β contact. The observation of both architectures suggests that homologous and heterologous lateral interfaces have comparable energetic stability, such that formation of a seam does not substantially compromise filament integrity. The coexistence of the two architectures may arise from the rapid polymerization of *Heim*–α/β-tubulin under the high concentrations used for in vitro assembly. Under physiological conditions, where assembly may be more tightly regulated, it is possible that a single architecture, likely the one-seam form, predominates.

### The *Heim–*α/β-tubulin subunit interactions

**Fig. 4.**
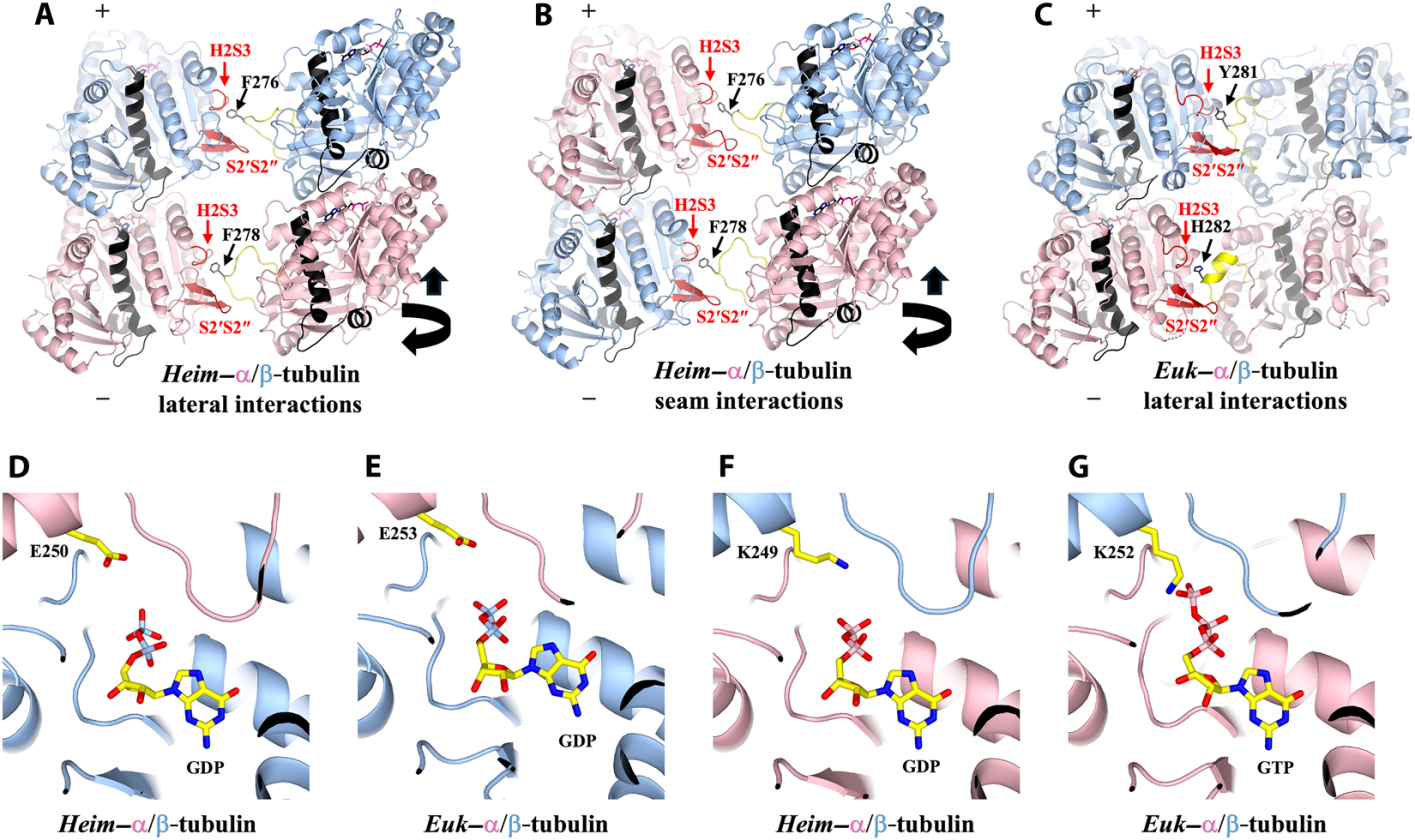
*Heim–*α/β-tubulin subunit interactions within the microtubule lattice. (**A** and **B**) Lateral interfaces between *Heim*–α-tubulin (pink) and *Heim–*β-tubulin (light blue) in the homologous lattice interaction (A) and the heterologous seam lateral interaction (B). Two subunits are shown in each protofilament (vertical), with a nucleotide (stick representation) positioned between longitudinally related subunits. The nucleotide-sensor motif is shown in black, spanning adjacent nucleotide-binding sites. The socket loops [H1-S2 (S2′S2″) and H2-S3] are shown in red, and the ball residues are shown in black and labeled within the M-loop (yellow; fig. S1), highlighting the conserved ball-and-socket lateral interaction. + and – indicate the plus and minus ends of the filaments, where the plus end is defined by the presence of the nucleotide-binding site. Thick arrows indicate the relative lateral offsets compared with the geometry of eukaryotic (*Euk*–) α/β-tubulin (**C**). (**D** to **G**) Comparison of the nucleotide-binding sites at the longitudinal interfaces in *Heim* and eukaryotic microtubules. The catalytic glutamate and noncatalytic lysine residues are shown.

To understand the molecular basis of *Heim*–α/β-tubule assembly, we examined the longitudinal and lateral interactions that stabilize the filament lattice. *Heim*–α/β-tubulin forms protofilaments composed of alternating *Heim*–α- and *Heim–*β-tubulin subunits, with a nucleotide sandwiched at each longitudinal interface, similar to *Odin*-*tubulin, *Loki–*α/β-tubulin, and eukaryotic α/β-tubulin. Lateral interactions between protofilaments are formed by Phe^276^ of *Heim–*β-tubulin and Phe^278^ of *Heim*–α-tubulin, which insert into a pocket formed by the H1-S2 (S2′-S2″) and H2-S3 loops of the neighboring subunit (Fig. 4, A and B). This arrangement forms a ball and socket–like interaction. The residues lining the pocket are either conserved or represent conservative substitutions between *Heim* α- and β-tubulins (fig. S1), consistent with the abilities of these interfaces to form homologous or heterologous lateral contacts.

A similar interaction is observed in eukaryotic microtubules, where residues with aromatic side chains (Tyr^281^ eukaryotic β-tubulin and His^282^ eukaryotic α-tubulin) into an equivalent pocket formed by the H1-S2 (S2′S2″) and H2-S3 loops (Fig. 4C and fig. S1). Thus, *Heim* tubules share a microtubule-like lattice with their eukaryotic counterparts. However, the lateral curvature and longitudinal rise for *Heim*–α/β-tubulin tubules differ substantially from those of eukaryotic microtubules. *Heim* tubules complete one revolution of the spiral in four subunits, whereas eukaryotic microtubules achieve a much more gradual curvature across 13 subunits. Despite these geometric differences, the ball-and-socket lateral interaction appears conserved between the 4- and 13-protofilament systems, suggesting a common structural principle underlying tubulin lattice assembly.

At the longitudinal interface between heterodimers, the catalytic glutamate of *Heim*–α-tubulin (Glu^250^) occupies a position similar to that of Glu^250^ in eukaryotic α-tubulin residue, pointing toward the β-phosphate of GDP in the neighboring β subunit (Fig. 4, D and E). As in other tubulin systems, this residue is positioned to orient the hydrolytic water for attack on the γ-phosphate of GTP, thereby promoting GTP hydrolysis during polymerization and generating GDP bound β-tubulin E-site, as we observe in the structure. This catalytic arrangement is conserved from *Odin*-*tubulin to eukaryotic microtubules (*11*). Unexpectedly, within the heterodimer interface of the *Heim*–α/β-tubulin protofilament, we observed density most consistent with GDP rather than GTP at the N-site, although weak density compatible with GTP was detectable at lower contour levels (fig. S9). This differs from eukaryotic α/β-tubulin, where the N-site is typically occupied by nonexchangeable GTP (Fig. 4, F and G). Because *Heim–*β-tubulin has the noncatalytic lysine (Lys^249^) corresponding to Lys^252^ in eukaryotic β-tubulin, hydrolysis at this site seems unlikely. Instead, we suggest that the N-site of the *Heim*–α/β-tubulin heterodimer may be more permissive, allowing either GDP or GTP to be incorporated during formation of the obligate heterodimer. Together, these observations indicate that both the catalytic architecture and the ball-and-socket lateral interface are conserved features of tubulin assembly, despite the markedly different protofilament number and lattice geometry of *Heim*–α/β-tubules. Hence, from now on, we refer to these structures as *Heim*–α/β-microtubules.

### Single *Heim–*α/β-microtubule dynamics observed by total internal reflection fluorescence microscopy

**Fig. 5.**
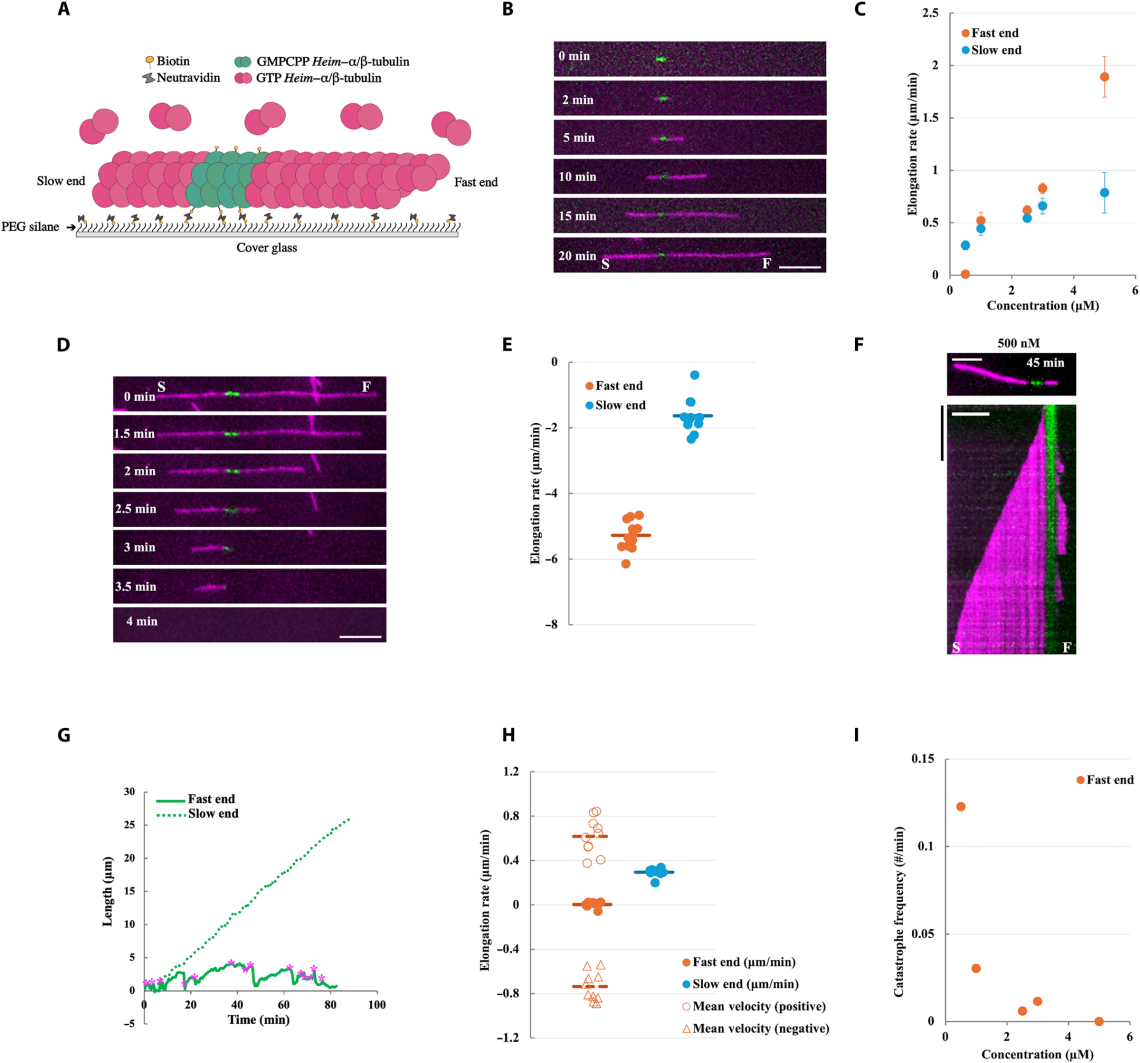
Single filament dynamics of *Heim–*α/β-microtubules visualized by TIRF microscopy. (**A**) TIRF assay schematic. GMPCPP-stabilized *Heim*–α/β-microtubule seeds were immobilized on biotin-PEG–coated coverslips via biotin-NeutrAvidin interactions. Fluorescent *Heim*–α/β-tubulin (magenta) polymerized from the seeds for real-time imaging. (**B**) Time-lapse images of *Heim*–α/β-tubulin polymerization (cropped from movie S1). Alexa Fluor 647–labeled *Heim*–α/β-tubulin (magenta) polymerized from immobilized GMPCPP-stabilized seeds labeled with Alexa Fluor 488 and biotin (green). *Heim*–α/β-tubulin concentration, 2.5 μM. Scale bar, 10 μm. (**C**) Polymerization rates of the fast and slow growing ends at various *Heim*–α/β-tubulin concentrations. At each concentration, six filaments were analyzed and the mean values were plotted. The error bars indicate the SD. (**D**) Time-lapse images of *Heim*–α/β-tubulin depolymerization (cropped from movie S2). Before the observation, *Heim*–α/β-microtubules were polymerized at 2.5 μM. Then, the free *Heim*–α/β-tubulin was removed by washing at 0 min. The GMPCPP seed (green) was also depolymerized. Scale bar, 10 μm. (**E**) Depolymerization rates of the fast and slow growing ends of *Heim*–α/β-microtubules. Time-averaged rates for individual filaments are shown (*n* = 12 filaments) with the mean values (solid lines). (**F** and **G**) Dynamic instability of *Heim*–α/β-microtubule observed at 500 nM. (F) Kymograph showing dynamic instability. Scale bars, 20 min (vertical) and 5 μm (horizontal). (G) Time courses of the fast and slow growing ends and catastrophe events (magenta asterisks). (**H**) Elongation rates of the fast and slow growing ends of *Heim*–α/β-microtubules showing dynamic instability (500 nM). Mean elongation rates of individual filaments time-averaged over the entire trajectory (closed circles), during the elongation (open circles) and shrinkage (open triangles) phases of the fast-growing ends. *n* = 10 filaments. Solid and dashed lines indicate the mean values. (**I**) Frequency of catastrophe events at the fast-growing ends, at various *Heim*–α/β-tubulin concentrations, detected from time course data. All the experiments were performed at 30°C.

To dissect the polymerization/depolymerization dynamics of *Heim*–α/β-microtubules, at the single-filament level, we used total internal reflection fluorescence (TIRF) microscopy (Fig. 5A). Short, guanosine-5’-[(α,β)-methylene]triphosphate (GMPCPP)-stabilized *Heim*–α/β-microtubule seeds (10% Alexa Fluor 488–labeled and 2.5% biotin-labeled) were immobilized on biotin–polyethylene glycol (PEG)–coated coverslips via biotin-NeutrAvidin interactions. Polymerization was initiated by introducing 2.5 μM soluble *Heim*–α/β-tubulin heterodimers (10% Alexa Fluor 647–labeled) in the presence of 1 mM GTP. Time-lapse imaging was performed at 30- to 60-s intervals for 30 min at 30°C. *Heim*-microtubules elongated from both ends of the seeds (Fig. 5B and movie S1). However, elongation rates differed significantly between the two ends (0.62 ± 0.05 μm/min versus 0.54 ± 0.01 μm/min; *P* < 0.01, two-sided Welch’s *t* test), demonstrating intrinsic kinetic polarity. Increasing *Heim*–α/β-tubulin concentration accentuated this asymmetry: The faster-growing end showed a strong concentration dependence, whereas the slower-growing end increased only modestly (Fig. 5C and fig. S10A). This behavior is characteristic of polar polymers.

We next examined depolymerization dynamics. After 30 min of growth at 2.5 μM, residual soluble *Heim*–α/β-tubulin heterodimers were rapidly washed out to initiate depolymerization. Time-lapse imaging captured rapid shortening from both ends (Fig. 5, D and E; movie S2; and fig. S10B), with one end consistently depolymerizing faster than the other, indicating preserved polarity during disassembly. At intermediate concentrations (0.5 to 1 μM), we observed stochastic switching between growth and shrinkage (movie S3). At 500 nM *Heim*–α/β-tubulin, one microtubule end elongated steadily, whereas the opposite end displayed a sawtooth-like length trajectory indicative of the dynamic instability observed for eukaryotic microtubules (Fig. 5, F and G, and fig. S10C). During growth phases, this dynamically switching end elongated faster than the continuously growing end (Fig. 5H). During catastrophe, the fast-growing ends shrank back to the initial GMPCPP-stabilized *Heim*–α/β-microtubule seeds, indicating that the entire GDP-bound segments depolymerized, but the GTP analog–bound subunits halted continued depolymerization. Catastrophe frequency decreased with increasing *Heim*–α/β-tubulin concentration and was nearly undetectable above 2.5 μM (Fig. 5I). Catastrophe events at the minus end were not detected under any tested condition. Together, these results demonstrate that *Heim*–α/β-microtubules exhibit intrinsic kinetic polarity, concentration-dependent asymmetry of elongation rates, and fast-growing end dynamic instability at intermediate concentrations, closely paralleling the core dynamic properties of eukaryotic microtubules.

### *Heim–*α/β-microtubule formation in liposomes

**Fig. 6.**
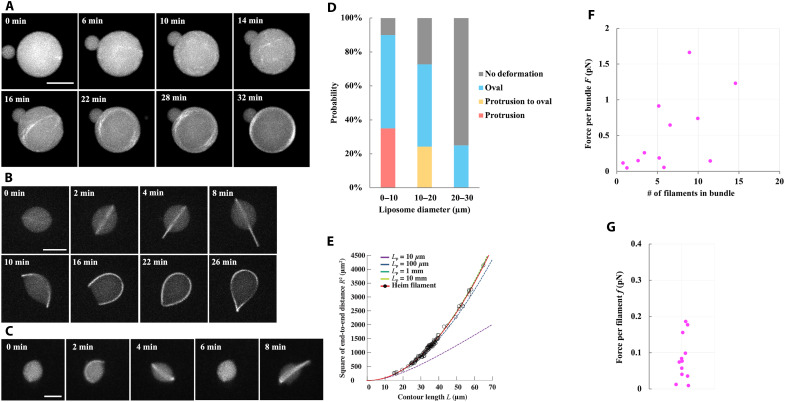
Dynamics of *Heim*–α/β-microtubules in liposomes visualized by confocal microscopy and estimation of mechanical force generated by polymerization. (**A** to **C**) Time-lapse images of liposomes encapsulated with *Heim*–α/β-tubulin observed under a confocal microscope. Polymerization of *Heim*–α/β-tubulin was induced inside liposomes by elevating the temperature from 4° to 37°C after the encapsulation. (A) Bundling of *Heim*–α/β-microtubules were observed inside a liposome ∼16 μm in diameter. Scale bar, 10 μm. (B) Membrane protrusion to oval transition induced by elongation of *Heim*–α/β-microtubule bundle inside a liposome ∼11 μm in diameter. Scale bar, 10 μm. (C) Formation of stable membrane protrusion by *Heim*–α/β-tubulin inside a liposome ∼5 μm in diameter. Scale bar, 5 μm. (**D**) Liposome morphology and the size dependence. Quantification of liposomes showing no deformation versus deformation with different shapes. 0 to 10 μm, *n* = 20 liposomes; 10 to 20 μm, *n* = 33 liposomes; 20 to 30 μm, *n* = 8 liposomes. (**E**) Persistence length analysis of *Heim*–α/β-microtubules. Relationship between contour length (*L*) and the square of end-to-end distance (*R*^2^) of individual filaments were used to estimate the persistence length (*L*_p_). Each open black circle represents experimental data (*n* = 105 filaments). The solid red line represents the fitted curve (coefficient of determination *R*^2^ = 0.994). The dashed lines represent theoretical curves with indicated *L*_p_ values. (**F** and **G**) Estimation of mechanical force generated (F) by bundles and (G) by single filaments of *Heim*–α/β-microtubules in liposomes.

Cellular studies of *Heim*–α/β-tubulin function are not possible since the native organism has yet to be isolated. To explore potential physiological roles, we therefore used an in vitro reconstitution approach, encapsulating purified *Heim*–α/β-tubulin heterodimers within lipid vesicles. Liposome encapsulation has previously been used to study how cytoskeletal polymers generate forces and reshape membranes (*19*–*21*). We encapsulated 5 μM *Heim*–α/β-tubulin (10% Alexa Fluor 647–labeled) into liposomes on ice and subsequently raised the temperature to 37°C under a confocal microscope to induce *Heim*–α/β-microtubule assembly. The osmotic pressure inside the liposomes was set slightly lower than that of the surrounding medium (Δ ≈ 40 mosmol), allowing the liposome to be deformable. *Heim*–α/β-microtubule structures appeared within ∼10 to 20 min after temperature increase. In many liposomes across various sizes (4 to 30 μm in diameter), *Heim*–α/β-microtubules spontaneously formed ring-shaped bundles near the equatorial plane that progressively fused to form thicker structures. These bundles moved along the inner membrane without causing global deformation of the liposome (Fig. 6A and movie S4). This behavior indicates that *Heim*–α/β-microtubules can self-bundle in the absence of accessory proteins, consistent with negative-stain electron microscopy observations showing bundle formation under identical buffer conditions (fig. S4B).

In a subset (two of eight) of large liposomes (20 to 30 μm in diameter), *Heim*–α/β-microtubules transiently assembled mitotic spindle-like structures before transforming into equatorial rings (fig. S11, A and B). During this intermediate stage, the liposome adopted an oval, rugby-ball-like shape (Fig. 6D and fig. S11A), which relaxed to a sphere as *Heim*–α/β-microtubules assembled into ring-shaped bundles. These observations suggest that *Heim*–α/β-microtubule assembly can generate mechanical forces capable of deforming membranes. Membrane deformation was more pronounced in intermediate-sized liposomes (10 to 20 μm in diameter), where >70% of liposomes (24 of 33) exhibited clear shape changes (Fig. 6, B and D, and movie S5). In ∼67% of these liposomes, ring-shaped bundles deformed the vesicle into an oval shape (Fig. 6D). In the remaining ∼33% of liposomes, bundles transiently generated membrane protrusions (Fig. 6B, 4 min). Continued elongation led to bundle buckling (Fig. 6B, 16 min), after which the liposomes relaxed to an oval shape (Fig. 6B, 22 min, and fig. S11A). Notably, in small liposomes (<10 μm in diameter), stable membrane protrusions were frequently observed (7 of 20 liposomes; 35%) and persisted for extended periods (Fig. 6C, fig. S11C, and movie S6). Although the cellular dimensions of the parent Heimdallarchaeales organism remain unknown, these observations indicate that *Heim*–α/β-microtubule assembly can generate localized forces sufficient to deform membranes, suggesting a potential role in shaping cellular membranes or generating protrusive structures in vivo.

### Mechanical properties of *Heim–*α/β-microtubules

To quantify the mechanical properties of *Heim*–α/β-microtubules, we measured their persistence length (*L*_p_), a fundamental parameter describing polymer bending rigidity (*22*). Shape analysis of individual *Heim*–α/β-microtubules observed under TIRF microscopy (*23*) yielded a persistence length of *L*_p_ = 481 μm (Fig. 6E). This value places *Heim*–α/β-microtubules among the stiffest known cytoskeletal polymers. However, they are still less rigid than eukaryotic microtubules, which exhibit persistence lengths of 1 to 10 mm (*24*–*26*). In contrast, eukaryotic F-actin is substantially more flexible, with an *L*_p_ = 9 to 20 μm (*23*, *27*), approximately 30-fold shorter than that of *Heim*–α/β-microtubules.

### Estimation of polymerization forces

Using this persistence length, we estimated the pushing forces generated by bundle elongation during membrane deformation (*28*). Immediately before and after membrane protrusion formation, bundles frequently exhibited buckling, allowing estimation of the critical force for buckling (*F*)

F=Nπ2κL2 (1)

where *N* is the number of *Heim*–α/β-microtubules in the bundle, κ is the bending rigidity of a single filament, and L is the contour length of the bundle (*29*). The bending rigidity is related to the persistence length by

$$L_{p}=\frac{\kappa }{k_{B}T}$$ (2)

where $k_{B}$ and T are the Boltzmann constant and temperature, respectively (*29*). Using the measured persistence length, we estimate as $\kappa =1.97\times 10^{-24}$ N^2^·m for a single *Heim*–α/β-microtubule.

We measured the contour length of buckled bundles *L* immediately after protrusion formation and estimated *N* by comparing bundle fluorescence intensity with that of single filaments. Substituting these values into Eq. 1 yielded estimated membrane-pushing forces *F* = 0.05 to 1.67 pN, with a mean of 0.5 pN (*n* = 12) (Fig. 6F). The estimated number of *Heim*–α/β-microtubules within protrusive bundles ranged from 1 to 15, with a mean of 6.3 filaments, confirming that *Heim*–α/β-microtubules assemble into bundles. Dividing total bundle force by *Heim*–α/β-microtubule number (*f* = *F*/*N*) gives an estimated force generated by polymerization of a single microtubule *f* = 0.08 ± 0.06 pN (means ± SD, *n* = 12) (Fig. 6G). This value is comparable to the force generated by polymerizing eukaryotic actin filaments (∼0.1 pN) (*30*) but is approximately 40- to 50-fold lower than forces reported for eukaryotic microtubules (3 to 4 pN) (*31*). We note that the force generated by *Heim*–α/β-tubulin polymerization is likely underestimated, as shorter bundles require higher forces to buckle (scaling as 1/*L*^2^; see Eq. 1). Because buckling was not detectable in many small liposomes, these potentially higher-force events were excluded from the analysis.

## DISCUSSION

Here, we describe a heterodimeric α/β-tubulin system from Heimdallarchaeales that assembles into microtubule-like tubules. Biochemical and structural analyses show that *Heim*–α/β-tubulin forms GTP-dependent protofilaments that assemble into four-protofilament microtubules with conserved longitudinal and lateral contacts similar to those observed in eukaryotic microtubules. Single-filament imaging reveals kinetic polarity and concentration-dependent dynamic instability, while liposome encapsulation demonstrates force generation. Together, these findings establish that Heimdallarchaeales tubulins form dynamic, microtubule-like polymers with key structural and functional properties of eukaryotic microtubules.

### Evolution

The evolutionary relationship between Asgard and eukaryotic tubulins remains unresolved. Phylogenetic analysis places the polymerizing *Heim* and *Loki* α- and β-tubulins as sister clades distinct from the eukaryotic α- and β-tubulin lineages (Fig. 1A), suggesting that α/β heterodimerization may have arisen independently in more than one lineage. The four-protofilament architecture observed here could therefore represent either an ancestral microtubule or a derived adaptation following horizontal gene transfer. The occurrence of reduced protofilament-number microtubules in bacterial BtubA/B systems and *Heim-* and *Loki*–α/β-microtubules supports a role for horizontal gene transfer in dissemination of simplified microtubules across prokaryotes. However, the presence of homopolymeric *Odin*-*tubulin, reduced protofilament-number microtubules in *Heim* and *Loki* lineages, and a predominantly 13-protofilament eukaryotic microtubules is also consistent with a diversification model. In this model, tubulins evolved from ancestral cell division proteins (FtsZ/CetZ), followed by gene duplication and catalytic specialization to generate α/β heterodimers that assemble into low protofilament-number microtubules, and subsequent expansion to higher protofilament numbers. These transitions likely occurred along the lineage leading from Asgard archaea to eukaryotes.

### Implications of the α/β-tubulin architecture

**Fig. 7.**
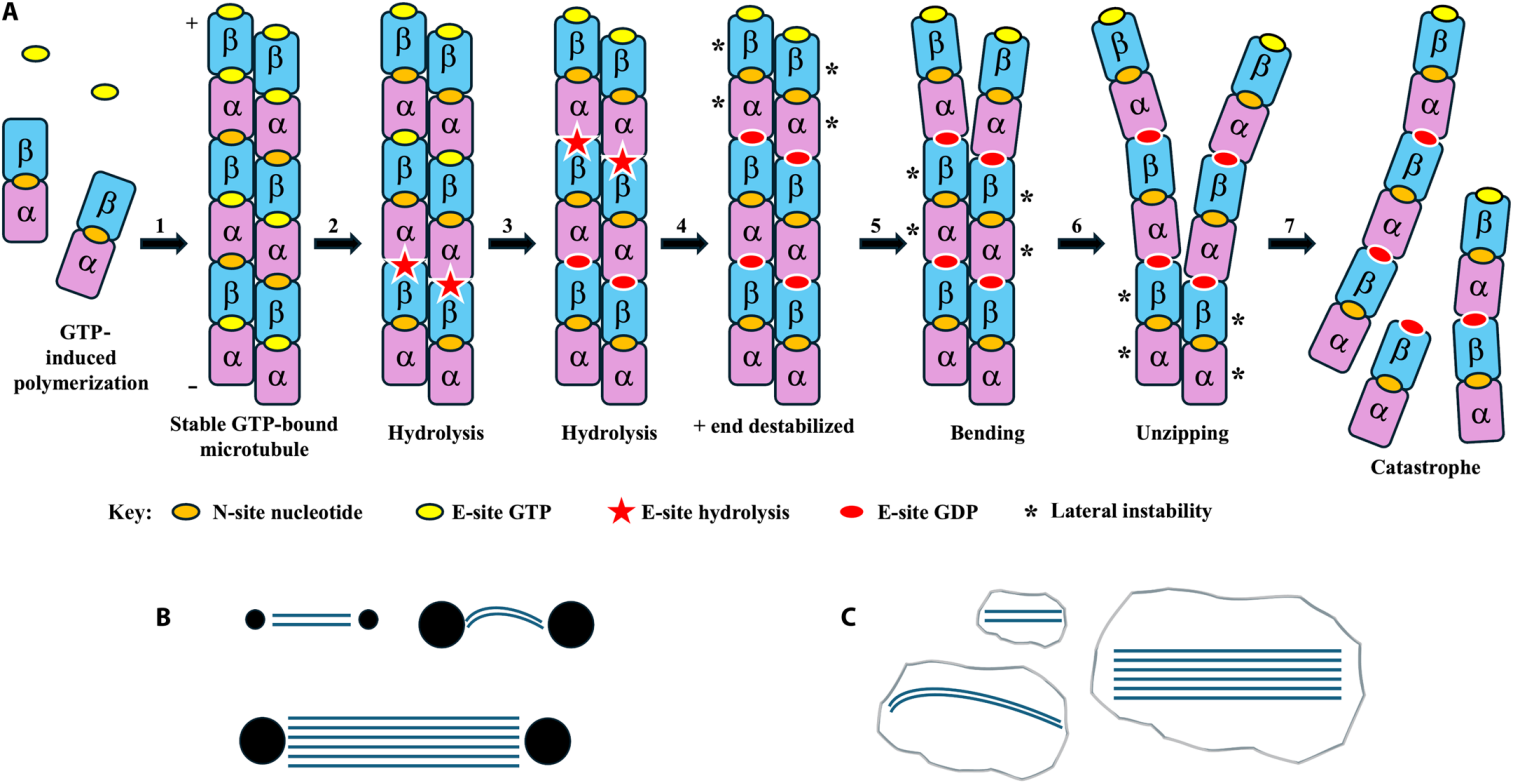
Models of *Heim–*α/β-microtubule plus-end dynamics and mechanical properties. (**A**) Model for polymerization and depolymerization of *Heim*–α/β-microtubules at the plus ends. For clarity, only two of the four protofilaments are shown. (1) *Heim*–α/β-tubulin heterodimers polymerize when GTP occupies the exchangeable site (E-site) in *Heim–*β-tubulin, while the nonexchangeable site (N-site) contains either GTP or GDP. (2 and 3) Following incorporation into the microtubule lattice, GTP hydrolysis at the E-site produces GDP; however, the protofilament remains straight because of stabilization by extensive lateral contacts. (4 and 5) Hydrolysis in the plus end–proximal *Heim–*β-tubulin subunits induces a conformational change that destabilizes the terminal subunits, which lack sufficient lateral contacts, causing the protofilament to curve away from the *Heim*–α/β-tubulin lattice. (6) This leads to a progressive loss of contacts through an unzipping mechanism, culminating in (7) catastrophe. (**B** and **C**) Schematic representations illustrating how tubule dimensions and protofilament number are adapted to mechanical load (B) and cellular dimensions (C). Larger circle indicates larger mechanical load. For simplicity, two and six protofilaments are drawn for *Heim*–α/β-tubulin and eukaryotic tubulins, respectively.

The repeated emergence of α/β heterodimers, in which only one subunit is catalytically active, suggests a functional advantage of this design. In the homopolymeric system of *Odin*-*tubulin, where all subunits can hydrolyze GTP, nucleotide hydrolysis drives cooperative conformational changes that drive rapid protofilament curvature (*11*). This effect is amplified by the nucleotide-sensor motif, which couples adjacent subunits and propagates bending along the protofilament. In contrast, incorporation of alternating nonhydrolyzing subunits disrupts this cooperativity, limiting the propagation of conformational changes and thereby stabilizing straight protofilaments. Although individual heterodimers remain intrinsically strained following GTP hydrolysis, this strain is buffered within the microtubule lattice by lateral interactions between neighboring protofilaments. At the plus end, however, terminal subunits lack sufficient lateral stabilization, allowing curvature to develop and promoting protofilament unzipping and catastrophe when subunit addition does not keep pace with hydrolysis. Thus, the α/β heterodimer architecture reduces longitudinal cooperativity in GTP hydrolysis–driven conformational changes, resulting in longer-lived, mechanically stable protofilaments while retaining the capacity for dynamic instability, providing a tunable balance between filament stability and dynamic turnover (Fig. 7A).

### Protofilament lateral contacts

In addition to the conserved α/β heterodimer architecture, the *Heim*–α/β-microtubule retains ball and socket–like interactions between neighboring protofilaments. This interaction motif appears inherently adaptable, permitting changes in protofilament number through relatively minor alterations at the lateral interface. This variability is evident across tubulin systems, where prokaryotic microtubules are composed of four or five protofilaments and eukaryotic microtubules can adopt architectures with fewer or greater protofilaments than the canonical 13-protofilament microtubule (*32*). These observations suggest that protofilament number is an evolutionarily tunable feature rather than a fixed property of tubulin polymers. We therefore considered the physical and cellular constraints that may govern protofilament number.

### Load and cell size

The persistence length of *Heim*–α/β-tubulin four-protofilament microtubules is ∼2- to 20-fold shorter than that of canonical 13-protofilament microtubules, consistent with the strong dependence of bending stiffness on cross-sectional geometry. Correspondingly, the polymerization force is estimated to be ∼40- to 50-fold lower. These differences suggest that increasing mechanical load (Fig. 7B) and cell size (Fig. 7C) would favor the evolution of higher protofilament numbers during eukaryogenesis. In particular, the emergence of tubulin-based flagella would require substantially stiffer filaments (*14*). Nevertheless, *Heim*–α/β-microtubules remain orders of magnitude stiffer than F-actin, indicating that even reduced protofilament architectures are well suited to bear compressive loads and span cellular distances. Consistent with this, our liposome experiments demonstrate that *Heim*–α/β-microtubule growth induces membrane protrusions, supporting a role for ancestral tubulins as force-generating cytoskeletal elements capable of shaping cellular membranes. However, the four-protofilament architecture appears limited in its ability to generate long protrusions (>10 μm), whereas LECA is estimated to have been substantially larger (∼2.5-fold) (*14*), suggesting evolutionary pressure during eukaryogenesis to increase protofilament number.

From the perspective of small Asgard cells, a reduced protofilament number may improve the efficiency of tubule assembly by conserving monomer resources. The maximum number of cell-spanning tubules that can be assembled scales as $\mathrm{cl}r^{2}/a$, where *c* is the tubulin concentration, *l* is the longitudinal length of the tubulin heterodimer, *a* is the protofilament number, and *r* is the radius of the cell. This relationship predicts that the number of available tubules is strongly limited in small cells (small *r*). Assuming intracellular tubulin concentrations comparable to those measured for eukaryotic tubulin in fission yeast (∼4 μM) and FtsZ in bacteria (∼3.5 μM) (*33*, *34*) and using *l* ≈ 8 nm for both eukaryotic and *Heim*–α/β-tubulin heterodimers, a 13-protofilament microtubule would be unable to form a cell-spanning structure in cells smaller than ∼0.8 μm in diameter due to monomer limitation. In contrast, a four-protofilament microtubule would still span cells as small as ∼0.4 μm in diameter.

Together, these considerations suggest that reduced protofilament number is advantageous for small cells with limited resources, whereas increased mechanical load, cell size, and the emergence of microtubule-based flagellar motility likely drove the evolution of higher protofilament numbers during eukaryogenesis. The canonical 13-protofilament microtubule was likely suited to the size and mechanical demands of LECA. As proposed for the actin cytoskeleton (*35*), protofilament number may have become effectively fixed because of the extensive network of interacting partners, including motors and nucleation proteins, as well as higher-order structures such as flagella, thereby constraining further structural variation during eukaryotic diversification.

## MATERIALS AND METHODS

### Phylogenetic analysis

Tubulin homologs were identified by BLAST searches of the NCBI nonredundant database (www.ncbi.nlm.nih.gov/) using *Odin*-*tubulin and representative eukaryotic tubulins as queries. Redundant and truncated sequences were removed, and representative sequences from Asgard lineages were selected for phylogenetic analysis. Structure-based multiple sequence alignment was performed in MAFFT using the DASH option, which incorporates structural information to guide alignment (*36*). A maximum likelihood phylogenetic tree was constructed in MEGA12 (*37*) using the LG (+F) substitution model (*38*), with 500 bootstrap replicates to assess branch support. Sequences included in the alignment shown in Fig. 1B correspond to proteins with available structures from *Ca.* Lokiarchaeum ossiferum [Protein Data Bank (PDB) codes: 9HXC and 9F6T], *Candidatus* Odinarchaeum yellowstonii (PDB code: 7EVE), and *Xenopus laevis* (eukaryotic tubulin; PDB code: 9G0P).

### Protein expression and purification

Codon-optimized genes encoding *Heim*–α-tubulin (MDH5401501.1) and *Heim–*β-tubulin (MDH5401500.1) were synthesized and cloned into pSY5, encoding an N-terminal 8×His-tag followed by a human rhinovirus (HRV) 3C protease cleavage site. *Heim–*β-tubulin was engineered with an additional N-terminal MAGEVVV sequence from a different predicted start codon to the annotated sequence (fig. S1). Plasmids were transformed into BL21(DE3) cells and expressed by induction with 0.3 mM isopropyl-β-d-thiogalactopyranoside (IPTG) at 15°C overnight. For heterodimer production, *Heim*–α-tubulin and *Heim–*β-tubulin were cloned into pRSFDuet for coexpression, with a 6×His-tag fused to the N terminus of *Heim*–α-tubulin. The plasmid was transformed into BL21(DE3) cells and induced with 0.3 mM IPTG at 15°C for 20 to 24 hours.

Cells were lysed in buffer containing 100 mM K-phosphate (pH 7.5), 150 mM NaCl, 0.5 mM MgSO_4_, and 20 mM imidazole using sonication (Sonifier 250, Branson). Lysates were clarified by centrifugation and applied to Ni–nitrilotriacetic acid resin (QIAGEN). After washing with five column volumes (CVs) of lysis buffer, the His-tag was removed by overnight HRV 3C protease cleavage at 4°C. Proteins were further purified by size exclusion chromatography (Superdex 200 Increase 10/300 GL, Cytiva) in Heim buffer [100 mM Pipes-KOH (pH 6.8), 150 mM NaCl, 0.5 mM MgSO_4_, and 0.5 mM EGTA]. Fractions were analyzed by SDS-PAGE and concentrated using 30-kDa molecular weight cutoff centrifugal filters.

Molecular weights of *Heim*–α-tubulin, *Heim–*β-tubulin, and the *Heim*–α/β-tubulin heterodimer were assessed by loading 6 μM protein onto the Superdex 200 Increase 10/300 GL column (Cytiva) with elution monitored at 280 nm and analyzed by SDS-PAGE.

### Light scattering

*Heim*–α/β-tubulin at varying concentrations was mixed with polymerization buffer [100 mM Pipes-KOH (pH 6.8), 0.5 mM MgSO_4_, 0.5 mM EGTA, 2 mM GTP, and 10% glycerol] and incubated at 37°C. Turbidity was monitored continuously at 340 nm using a SpectraMax i3 (Molecular Devices) plate reader using 384-well clear, flat-bottom plates (781096, Greiner Bio-one). GDP controls contained 2 mM GDP in place of GTP.

### Pi release assay

GTP hydrolysis was measured using the EnzChek Phosphate Assay Kit (E-6646, Thermo Fisher Scientific) (*17*). Reactions containing 15 μM *Heim*–α/β-tubulin in 100 mM Pipes-KOH (pH 6.8), 150 mM NaCl, 0.5 mM MgSO_4_, and 0.5 mM EGTA were mixed with 1 mM GTP and the EnzChek detection reagent. Reactions were incubated at 37°C, and Pi release was monitored at 360 nm using a plate reader (SpectraMax i3, Molecular Devices) with 96-well clear, flat-bottom plates (92696, Techno Plastic Products). Phosphate standards (0 to 100 μM) were used for calibration (fig. S3C). Contribution of polymerization-induced increase in light scattering at 360 nm was confirmed to be <10% of total signal.

### Fluorescence labeling of *Heim–*α/β-tubulin

*Heim*–α/β-tubulin was polymerized for 30 min at 37°C and labeled with Alexa Fluor 488 *N*-hydroxysuccinimide (NHS) ester (A20000, Invitrogen), Alexa Fluor 647 NHS ester (A22287, Invitrogen), or NHS-PEG-biotin (QBD10200, Sigma-Aldrich) at a 1:1 dye:tubulin molar ratio for 30 min at 37°C. Reactions were quenched with 10 mM tris-HCl (pH 7.2) for 5 min at 37°C. Labeled microtubules were pelleted (541,000*g* for 10 min at 37°C; TLA100.3 rotor, Beckman), depolymerized on ice in buffer containing GDP, and dialyzed overnight at 4°C to remove unincorporated dye and nucleotide. Aggregates were removed by ultracentrifugation (436,000*g* for 10 min at 4°C). Labeled monomers were snap frozen in liquid nitrogen and stored at −80°C.

### Preparation of GMPCPP-stabilized seeds

Seeds were prepared through 3 cycles of polymerization and depolymerization. For each cycle, unlabeled, Alexa Fluor 488–labeled (10%), and biotinylated (2.5%) *Heim*–α/β-tubulin was polymerized in the presence of 1 mM GMPCPP (NU-405, Jena Bioscience) for 60 min at 37°C. Microtubules were pelleted (436,000*g* for 10 min at 37°C) and depolymerized on ice for 30 min, and the cycle was repeated twice more. Final pellets were resuspended in warm Heim buffer, aliquoted, snap frozen, and stored at −80°C. Before use, frozen seeds were quickly thawed in a 37°C heat block.

### Flow chamber preparation

Flow chambers were constructed using a biotin-functionalized coverslip and a PEG-passivated coverslip, as previously described (*39*). Briefly, coverslips (22 mm by 22 mm, thickness no. 1S, noncoated, Matsunami) were sonicated in 1 N KOH for 15 min, washed 10 times with Milli-Q water, sonicated again in Milli-Q water for 15 min, and washed 10 times with Milli-Q water. The coverslips were dried with a nitrogen gas stream and cleaned in a plasma cleaner (PDC-32G, Harrick Plasma) for 5 min, followed by heating at 150°C for 10 min. The surface was silanized with 3-aminopropyltriethoxysilane (93-1402, Wako) for 20 min at room temperature. Coverslips were then washed 10 times with Milli-Q water, dried with nitrogen gas, and incubated with NHS-PEG (200 mg/ml; ME-050TS, Sun Bright) with or without NHS-PEG-biotin (1 mg/ml; BI-050TS, Sun Bright) for 90 min at room temperature to generate PEG-biotin–coated or PEG-coated glass. Last, coverslips were washed 10 times with Milli-Q water, dried quickly with nitrogen gas, and air dried completely on a clean bench for 30 min. Flow chambers were assembled by placing double-sided tape (thickness, 0.3 mm) between a PEG-biotin–coated glass (bottom) and a PEG-coated glass (top). The flow chambers were stored in a vacuum desiccator at below 0.08 MPa to prevent adsorption of dust until just before use.

### Assembly, disassembly, and dynamic instability observation

To immobilize tubules on a coverslip for imaging, we used a biotin-avidin system (*40*, *41*). First, neutravidin (1 mg/ml; 31000, Pierce) in Heim buffer was loaded on the flow chamber and incubated for 5 min to immobilize Neutravidin on the PEG-biotin–coated glass surface. After the incubation, five CVs of Heim buffer were flowed to wash out free neutravidin. Next, 0.5 μM GMPCPP *Heim*–α/β-microtubule seeds was loaded into the chamber and incubated for 2 min, followed by washing with 5 CVs of Heim buffer. The glass surface was then blocked with TIRF buffer [100 mM Pipes-KOH (pH 6.8), 0.5 mM MgSO_4_, 0.5 mM EGTA, 1% Pluronic F-127, 1 mM dithiothreitol (DTT), casein (1 mg/ml), glucose (4.5 mg/ml), glucose oxidase (0.3 mg/ml), catalase (25 μg/ml), and 0.2% methylcellulose (1500 cP)] for 2 min. Last, various concentrations of *Heim*–α/β-tubulin (10% Alexa Fluor 647–labeled) in TIRF buffer containing 1 mM GTP were flowed into the flow chamber and sealed with Valap, and the polymerization and dynamic instability were observed under a microscope. For the observation of the depolymerization process, *Heim*–α/β-tubulin was polymerized from the seeds at 37°C for 30 min as described above, and then the chamber was quickly washed by TIRF buffer containing 1 mM GTP to remove free *Heim*–α/β-tubulin.

### TIRF microscopy

Single-filament dynamics of *Heim*–α/β-microtubules were observed using a custom-built TIRF microscope (IX71, Olympus) equipped with a 60× objective (UPlanApo TIRF 60×/1.50 Oil HR, Olympus), an electron-multiplying charge-coupled device (EMCCD) camera (iXon3 897, Andor Technology), and excitation lasers (488 nm: OBIS 488-60-LS and 640 nm: OBIS 640-100-LX, Coherent). To reduce background noise and improve fluorescence signal uniformity, pseudo-annular illumination was generated using two-axis galvo mirrors (GVS212/M, Thorlabs) operating at 100 Hz and controlled by a function generator (FGX-2220, Texio). The sample temperature was maintained at 30°C during observation using a custom-built heat block through circulation of temperature-controlled water connected to a water bath (NCB-1220, EYELA).

### Liposome experiment

#### Lipid-oil mixture preparation

The lipid-oil mixture was prepared as previously described (*42*, *43*). Briefly, l-α-phosphatidylcholine from chicken egg yolk (EggPC; 840051P, Avanti Polar Lipids) and 1,2-dioleoyl-*sn*-glycero-3-phosphatidylglycerol (840475P, Avanti Polar Lipids) were mixed at a molar ratio of 85:15 in chloroform. The solvent was evaporated under a nitrogen stream, further dried under vacuum to form a thin film, and then resuspended in mineral oil (M8410, Sigma-Aldrich) at a final concentration of 5 mM. The lipid-oil mixture was heated to 80°C for 30 min and sonicated at 60°C for 90 min to dissolve the phospholipids completely.

#### Protein encapsulation into liposomes

For encapsulation, varying concentrations of *Heim*–α/β-tubulin were combined with the lipid-oil emulsion, as previously described (*44*). First, 100 μl of the lipid-oil mixture was gently placed on top of the outer solution [100 mM Pipes-KOH (pH 6.8), 250 mM glucose, 0.5 mM MgSO_4_, 0.5 mM EGTA, 1 mM GTP, 10 mM DTT, and bovine serum albumin (0.2 mg/ml)] and incubated on ice for 5 min. Meanwhile, 5 μM *Heim*–α/β-tubulin (10% Alexa Fluor 647–labeled) was mixed with the inner solution [100 mM Pipes-KOH (pH 6.8), 150 mM sucrose, 0.5 mM MgSO_4_, 0.5 mM EGTA, 1 mM GTP, 10 mM DTT, and 2 mM Trolox]. Then, 2 μl of this mixture was pipetted into 100 μl of the chilled lipid-oil mixture and vortexed for 10 s to form cell-sized droplets. The emulsion was gently placed onto the lipid-oil layer over the outer solution and centrifuged at 12,000*g* for 60 s at 4°C to transform the droplets into liposomes. The osmotic pressures of the inner (457 mosmol) and outer solutions (497 mosmol) were measured using a micro-osmometer (Fiske 210, Advanced Instruments).

#### Flow chamber for liposome observation

The flow chamber for liposome observation was assembled by placing two strips of double-sided tape (thickness, 0.3 mm) onto a coverslip (60 mm by 24 mm, thickness no. 1, NEO, Matsunami) and sealing with a top coverslip (18 mm by 18 mm, Matsunami). The liposome solution was loaded into the chamber and sealed with Valap.

#### Confocal microscopy

Confocal fluorescence images of the liposomes were observed by an inverted microscope (IX73, Olympus) equipped with a ×60 objective (UPlanSApo, numerical aperture: 1.30 sil, Olympus), a confocal scanner unit (CSU-X1, Yokogawa), and an electron-multiplying CCD camera (iXon3 897, Andor Technology). The sample temperature was maintained at 37°C by a custom-built heat block connected to a water bath (AB-1600, ATTO).

### Single filament dynamics analysis

The two ends of individual microtubules were manually tracked frame by frame using Fiji/ImageJ. Stage drift was manually corrected before analysis. Catastrophe events were defined as microtubule shrinkage exceeding 0.5 μm within 2 min after the elongation rate switched from positive to negative.

### Persistence length analysis

The persistence length of *Heim*–α/β-microtubules was estimated as previously described (*23*). Briefly, we manually measured the contour length *L* and end-to-end distance *R* of each filament using Fiji/ImageJ. Assuming that *R*^2^ and *L* follow the following equation (*22*)

$$\langle R^{2}\rangle =4{L_{p}}^{2}\;\left[\text{2exp}\left(–L/2L_{p}\right)–2+L/L_{p}\right]$$ (3)

the persistence length *L*_p_ of *Heim*–α/β-microtubules was estimated by fitting this equation to the experimental data.

### Liposome analysis

Liposomes were analyzed using Fiji/ImageJ. Maximum intensity projections of the Alexa Fluor 647 channel were generated, and liposome diameters were measured manually. Bundle length and fluorescence intensity were also measured manually, and background signals were subtracted. To estimate the fluorescence intensity of single microtubules, *Heim*–α/β-microtubules were polymerized following the TIRF assay protocol. Images were acquired using the same experimental setup as in the liposome experiments. Background signals were subtracted, and the fluorescence intensity of each *Heim*–α/β-microtubule was measured (*n* = 20 filaments).

### Negative staining

Negative-stain electron microscopy was performed as described previously (*11*). Briefly, *Heim*–α/β-tubulin heterodimer was diluted to the indicated concentrations in polymerization buffer [100 mM Pipes-KOH (pH 6.8), 0.5 mM MgSO_4_, 0.5 mM EGTA, and 10% glycerol] supplemented with 2 mM GTP and incubated at 37°C for 5 to 20 min (see fig. S4). For optimization of cryo-EM conditions, polymerization buffer was supplemented with 10 mM potassium phosphate (pH 6.8). A 2.5-μl aliquot was applied to glow-discharged STEM100Cu elastic carbon grids (Ohkenshoji Co. Ltd.) and allowed to adsorb for 1 min. Grids were blotted and stained with 5 μl of 2% (w/v) uranyl acetate for 1 min, blotted again, and air-dried overnight. Images were recorded using a H-7600 transmission electron microscope (Hitachi High-Tech) equipped with an Orius SC200D CCD camera (Gatan).

### Cryo-EM grid preparation

*Heim*–α/β-tubulin heterodimer (50 μM) was polymerized at 37°C for 10 min by addition of prewarmed K-Pipes buffer supplemented with 10 mM potassium phosphate and 2 mM GTP (Sigma-Aldrich), using conditions adapted from protocols for eukaryotic tubulin and OdinTubulin (*11*, *45*). A 2.1-μl aliquot was applied to glow-discharged molybdenum R1.2/1.3 or R2/2 200-mesh holey carbon grids (Quantifoil). Grids were blotted for 1.5 to 2.5 s at room temperature (90% humidity) and plunge-frozen using an EM GP plunge freezer (Leica).

### Cryo-EM data acquisition

Vitrified molybdenum grids were screened and imaged on a Titan Krios G4 transmission electron microscope (Thermo Fisher Scientific) operated at 300 kV and equipped with a Falcon 4i direct electron detector at the Research Institute for Interdisciplinary Sciences, Okayama University. Data were collected at ×165,000 magnification in counting mode with a calibrated pixel size of 0.75294 Å. Movies were recorded over 3.78 s (129 frames) with a total dose of 50 e^−^/Å^2^ and a defocus range of −0.6 to −1.9 μm using EPU software. A total of 4213 movies were collected.

### Cryo-EM image processing

Raw movies were processed and corrected for motion in RELION 5.0 (*46*), and CTF (Contrast Transfer Function) parameters were estimated using CTFFIND-4.1 (*47*). Micrographs with reliable CTF fits were selected for further analysis. Helical segments were initially manually picked to generate 2D class averages used as templates for Topaz-based automated picking. A total of 2,341,375 segments were extracted with 4 × 4 binning (box size, 128 pixels by 128 pixels) and subjected to 2D classification. From these, 1,048,149 particles corresponding to tubule-like classes were selected. Initial helical parameters were estimated from the 2D class averages, and an empty cylindrical model was generated using the RELION helix toolbox. After iterative 2D and 3D classification, 803,537 particles were retained for refinement.

3D refinement revealed discrimination between α and β subunits (fig. S7A). The C-terminal extension of the β subunit appeared as blurred density at low contour levels. Subsequent 3D classification without alignment identified distinct seam configurations (fig. S7B). The averaged density revealed four strands containing a single seam. On the basis of this map, eight templates (fig. S7C) were generated, representing four possible seam positions and two possible α-β arrangements. Another round of 3D classification without alignment was then performed using these templates. Particles were reassigned to ensure consistent seam and α/β orientation by editing STAR files based on class assignments, followed by further refinement (*48*). 3D refinements were then carried out using the edited parameters as starting values (fig. S7D). After refinement, α-β discrimination remained relatively poor in one strand located opposite the seam. Therefore, particles were separated into two classes by 3D classification using a mask encompassing this strand (fig. S7E). One class contained 376,876 particles with a single seam, whereas the other contained 87,877 particles exhibiting three seams. Each class was further processed by 3D refinement and additional 3D classification. The single-seam structure was subsequently improved by CTF refinement and Bayesian polishing. The final reconstruction of the single-seam filament was obtained from 223,429 particles at a resolution of 2.8 Å (fig. S7F), with final helical parameters of *dz* = 83.9 Å and *d*φ = 0.80°. Averaging the four strands in real space yielded a single-strand map at 2.4-Å resolution. The final reconstruction of the three-seam filament (fig. S7G) was obtained from 71,307 particles at a resolution of 3.9 Å, with helical parameters of *dz* = 83.9 Å and *d*φ = 0.73°.

### Modeling

An initial atomic model for the single-seam reconstruction was generated using AlphaFold3 (*49*). The model was refined against the 2.4-Å single-strand density using ISOLDE in ChimeraX (*50*, *51*), followed by iterative rebuilding in COOT (*52*) and real-space refinement in Phenix (*53*). Interface residues were modeled using the full single-seam reconstruction 2.8-Å map. The three-seam model was generated by rigid-body fitting of the refined single-seam subunit model, followed by manual adjustment. Cryo-EM data collection, processing, and structure statistics are summarized in table S1.
