# Supplementary material for: Structure and dynamics of a four-protofilament microtubule from Heimdallarchaeales α/β-tubulin
Source: Sci Adv. 2026 Jul 15;12(29):eaeh4305. doi: 10.1126/sciadv.aeh4305 (PMC13371889; doi:10.1126/sciadv.aeh4305)
Supplement: Supplementary file 1 — Figs. S1 to S11 Table S1 Legends for movies S1 to S7 [file sciadv.aeh4305_sm.pdf]

Science Advances
AAAS

Supplementary Materials for

Structure and dynamics of a four-protofilament microtubule from Heimdallarchaeales α/β-tubulin

Linh T. Tran *et al.*

Corresponding author: Akihiro Narita, narita.akihiro.x8@f.mail.nagoya-u.ac.jp;
Makito Miyazaki, makito.miyazaki@riken.jp; Robert C. Robinson, robert.b@vistec.ac.th

Sci. Adv. **12**, eaeh4305 (2026)
DOI: 10.1126/sciadv.aeh4305

The PDF file includes:

Figs. S1 to S11
Table S1
Legends for movies S1 to S7

Other Supplementary Material for this manuscript includes the following:

Movies S1 to S7

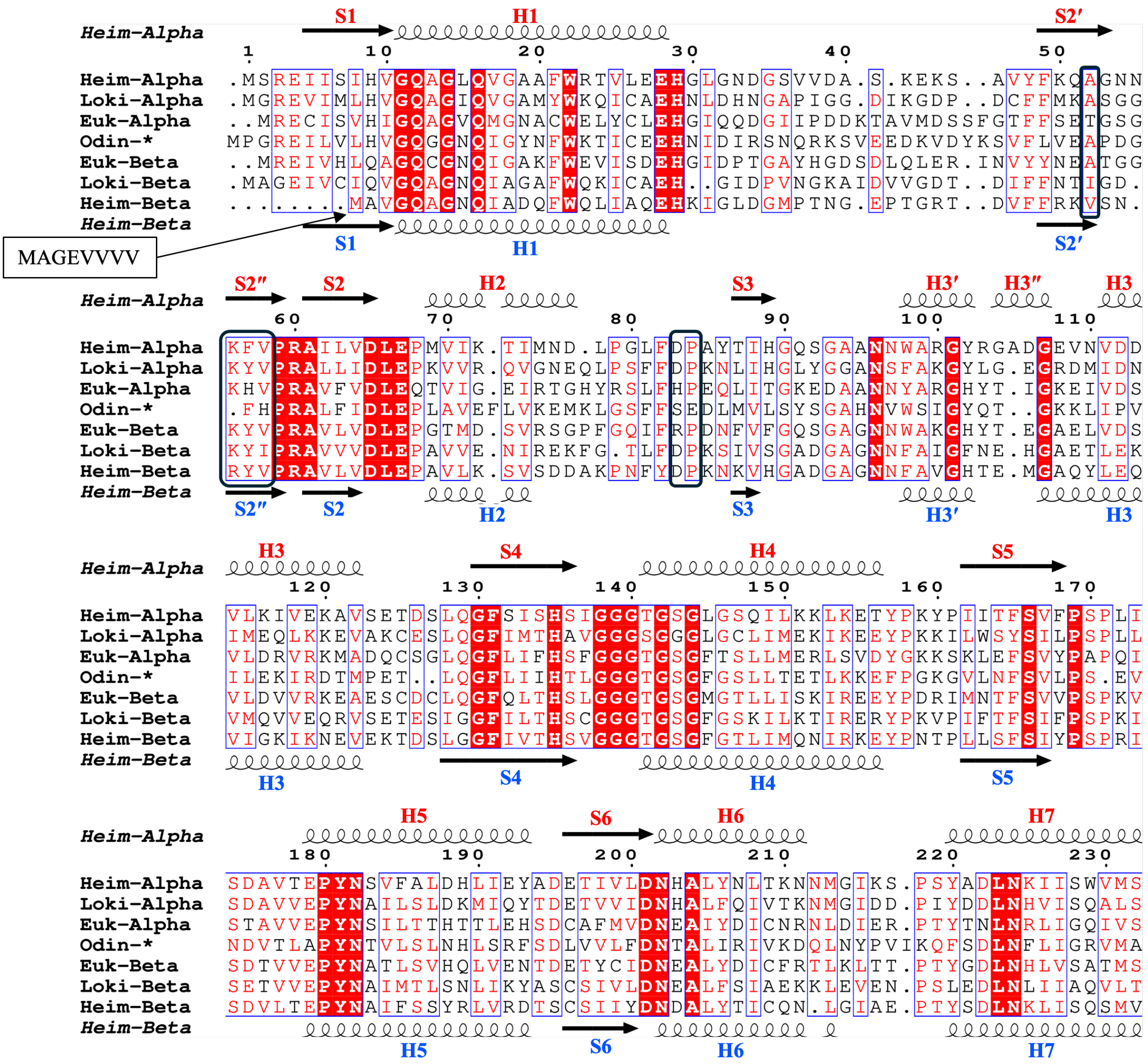

Fig. S1. Tubulin multiple sequence alignment. The alignment is shown with the secondary structure annotations above (*Heim*-α-tubulin) and below (*Heim*-β-tubulin) the alignment. Strands (S) and helices (H) are numbered as for eukaryotic tubulins. The boxed sequence with associated arrow shows the N-terminal residues arising from the alternative start site (used in this study) other than that from the annotated sequence (MDH5401500.1). Boxed amino acids indicate sequences from S2′S2″ (within the H1-S2 linker) and the H2-S3 linker that act as the "socket" in the ball-and-socket lateral interactions. The alignment is continued on the next page.

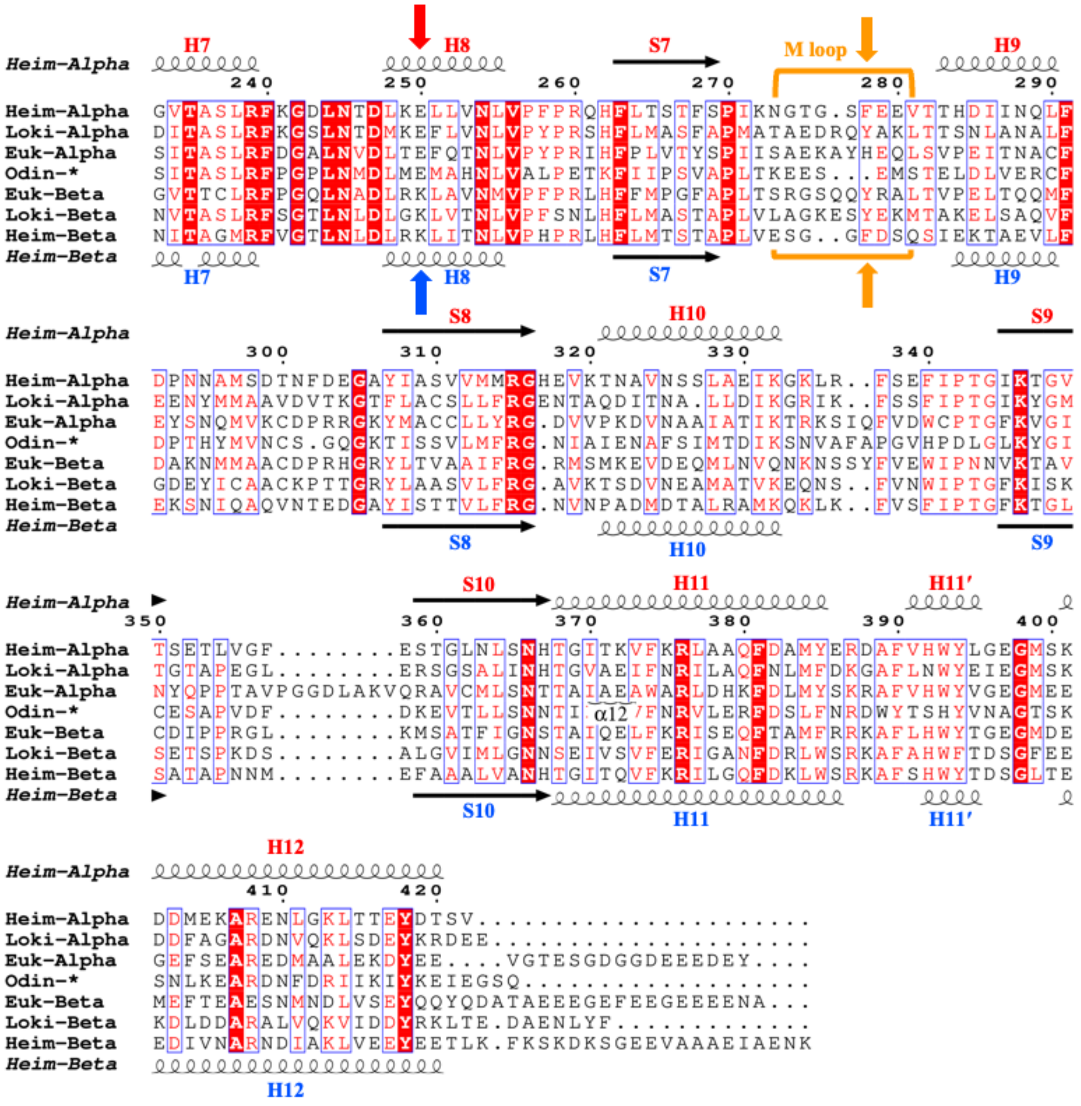

Fig. S1. Continued. Red and blue arrows indicate tubulins with a catalytic glutamate or non-catalytic lysine residue, respectively. Orange arrows indicate residues from the M-loop, which contain a ring system (Tyr, Phe or His), that act as the "ball" in the ball-and-socket lateral interactions.

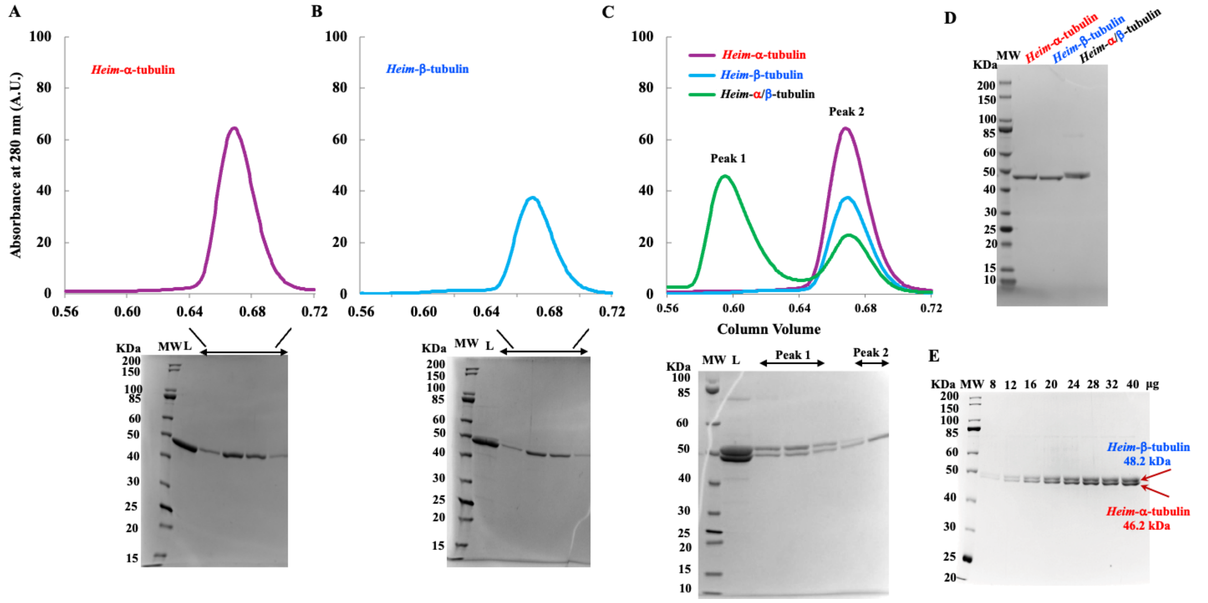

Fig. S2. *Heim*-α/β-tubulin protein characterization profiles. Gel filtration profiles of **(A)** *Heim*-α-tubulin, **(B)** *Heim*-β-tubulin, and **(C)** *Heim*-α/β-tubulin. In **(C),** the profile of *Heim*-α/β-tubulin (**Fig. 2A**) is overlayed with *Heim*-α-tubulin and *Heim*-β-tubulin. A.U., arbitrary units. SDS-PAGE gel images are shown below the profiles. **(D)** SDS-PAGE gel showing the single proteins and the complex. **(E)** High resolution SDS-PAGE minigel demonstrating the separation of *Heim*-α-tubulin and *Heim*-β-tubulin subunits based on differences in molecular size. Increasing amounts of purified *Heim*-α/β-tubulin complex were loaded. The doublet corresponds to *Heim*-α-tubulin (lower band) and *Heim*-β-tubulin (upper band) subunits.

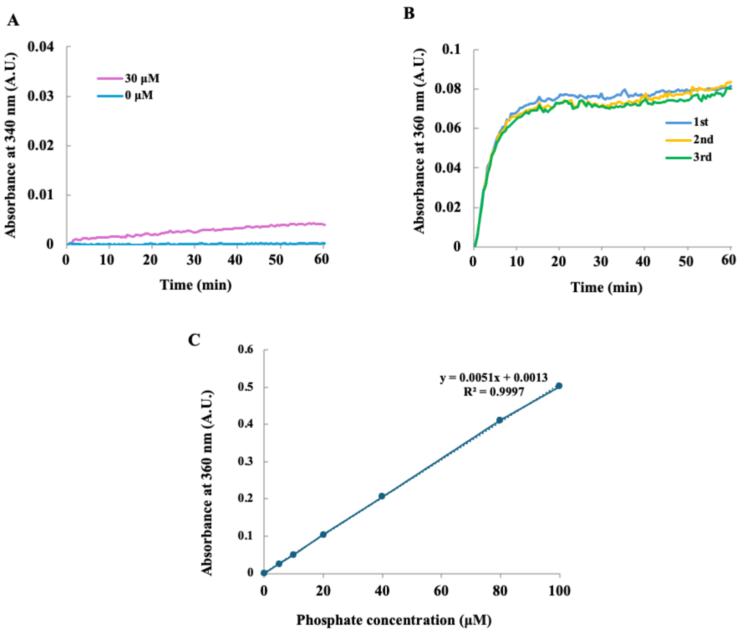

Fig. S3. ***Heim*-α/β-tubulin biochemical properties. (A)** Polymerization time course of *Heim*-α/β-tubulin (0 or 30 µM) in the presence of 2 mM GDP at 37 ºC. The polymerization activity was monitored by measuring absorbance at 340 nm. (**B**) Raw data for Pi release detection assays shown in Fig. 2C (15 µM) and fig. S3B (20 µM). **(C)** Standard curve of EnzChek detection reagent used to quantify the GTP hydrolysis rate of *Heim*-α/β-tubulin. Absorbance at 360 nm was measured at various Pi concentrations. A.U., arbitrary units.

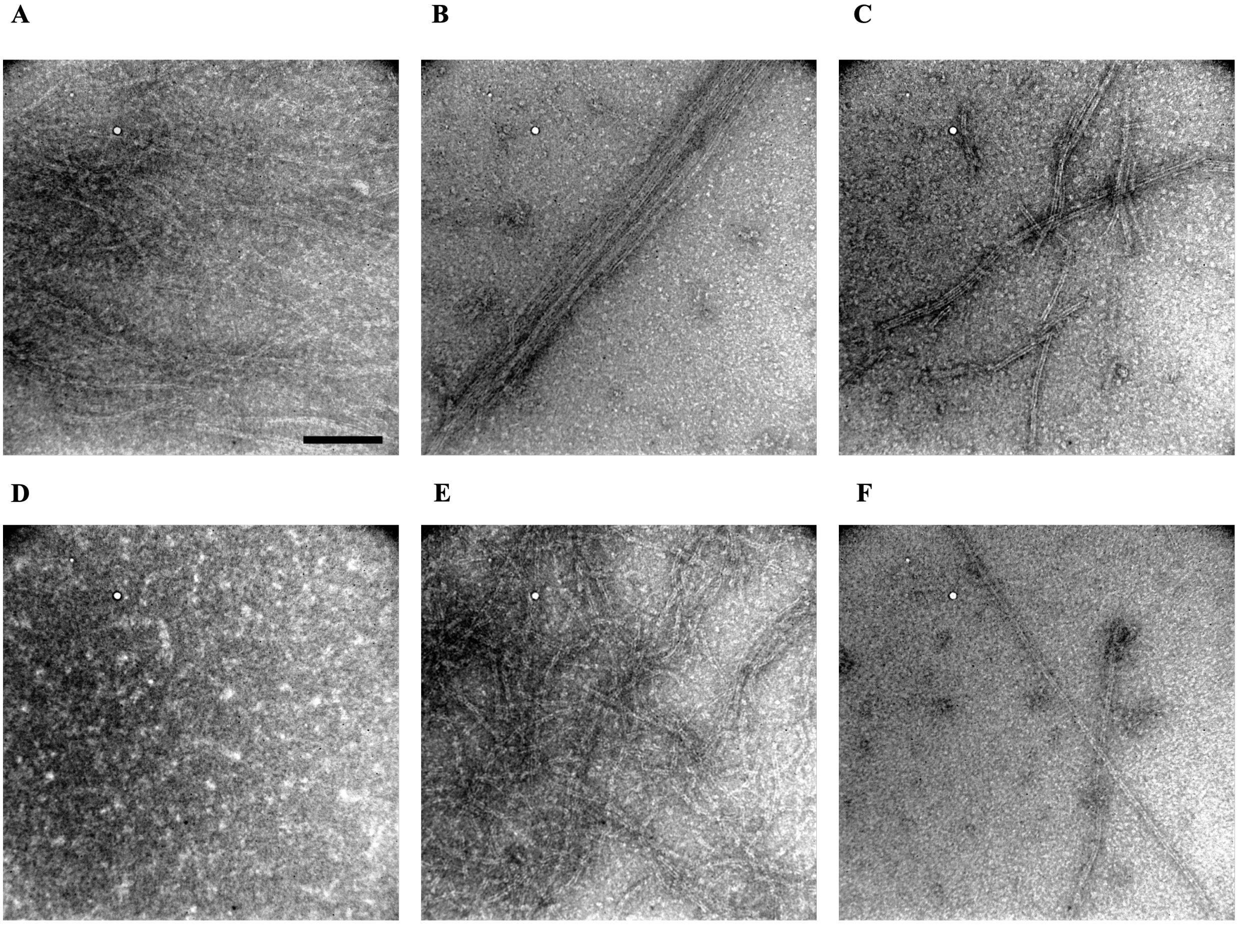

Fig. S4. *Heim*-α/β-tubulin filaments observed by electron microscopy of negatively stained samples. (**A** to **F**) *Heim*-α/β-tubulin was incubated at 37 °C in polymerization buffer containing 2 mM GTP for (**A** to **C**) 10 min, (**D** and **E**) 20 min or (**F**) 5 min. (**A**) Undiluted 10 µM *Heim*-α/β-tubulin. (**B**) Two-fold diluted 10 µM *Heim*-α/β-tubulin. Bundled filaments were observed. (**C**) Two-fold diluted 10 µM *Heim*-α/β-tubulin. Both short bundled and single filaments were observed. (**D**) Undiluted 20 µM *Heim*-α/β-tubulin. High filament density. (**E**) Two-fold diluted 20 µM *Heim*-α/β-tubulin. Lower filament density. Curved filaments were observed. (**F**) Undiluted 30 µM *Heim*-α/β-tubulin. Both single and bundled filaments were observed.

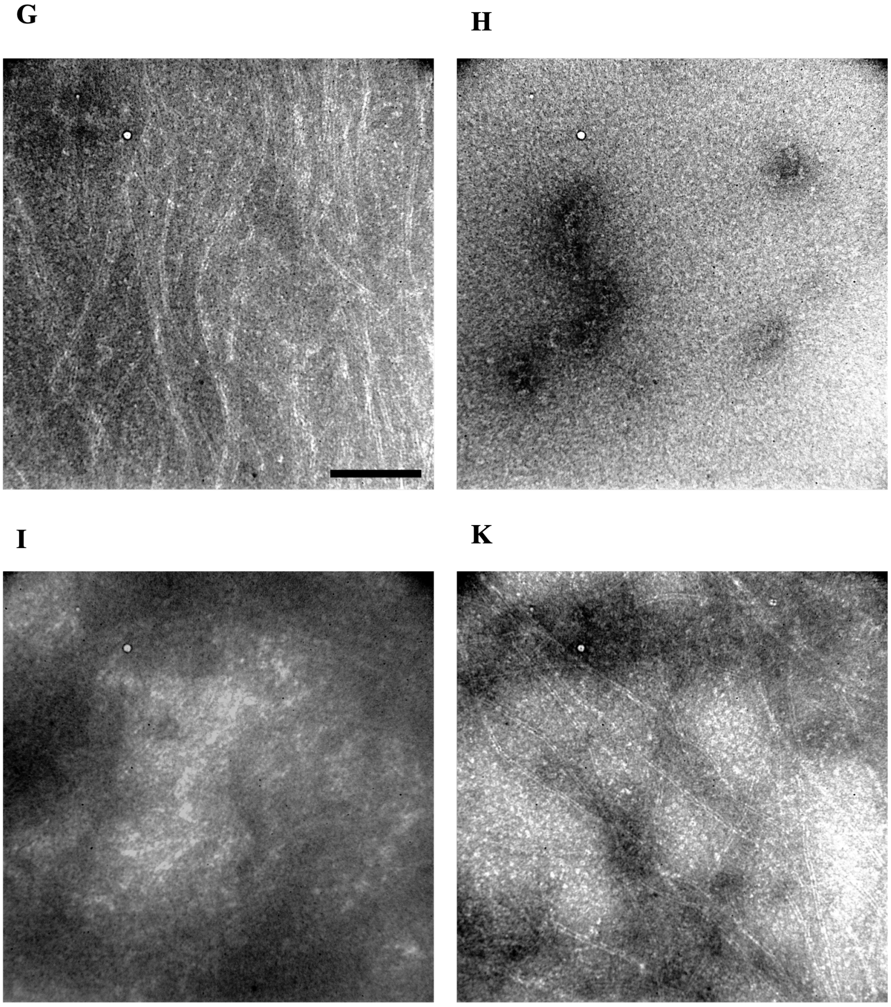

Fig. S4 continued. (**G** to **K**) *Heim*-α/β-tubulin was incubated at 37 °C for 10 min in polymerization buffer containing 10 mM potassium phosphate buffer (pH 6.8). (**G**) Undiluted 10 µM *Heim*-α/β-tubulin with 2 mM GTP. (**H**) Undiluted 10 µM *Heim*-α/β-tubulin with 2 mM GDP. (**I**) Undiluted 50 µM *Heim*-α/β-tubulin with 2 mM GTP. (**K**) Two-fold diluted 50 µM *Heim*-α/β-tubulin with 2 mM GTP showing straight, long filaments with high density, used for cryo-EM sample preparation. Scale bars, 200 nm.

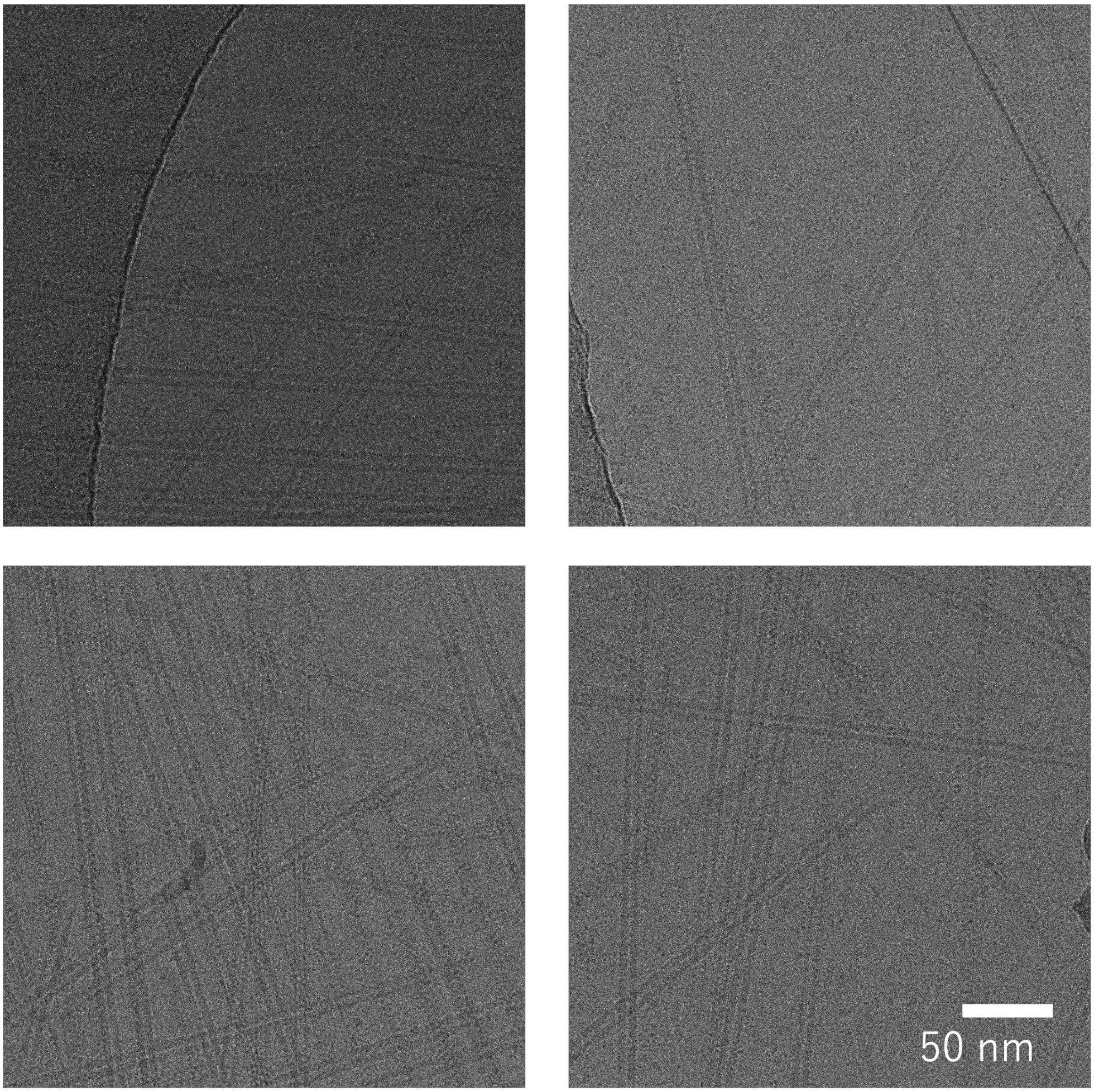

Fig. S5. Cryo-EM images of *Heim*-α/β-tubulin filaments. Straight filaments observed by cryo-EM. Scale bar, 50 nm.

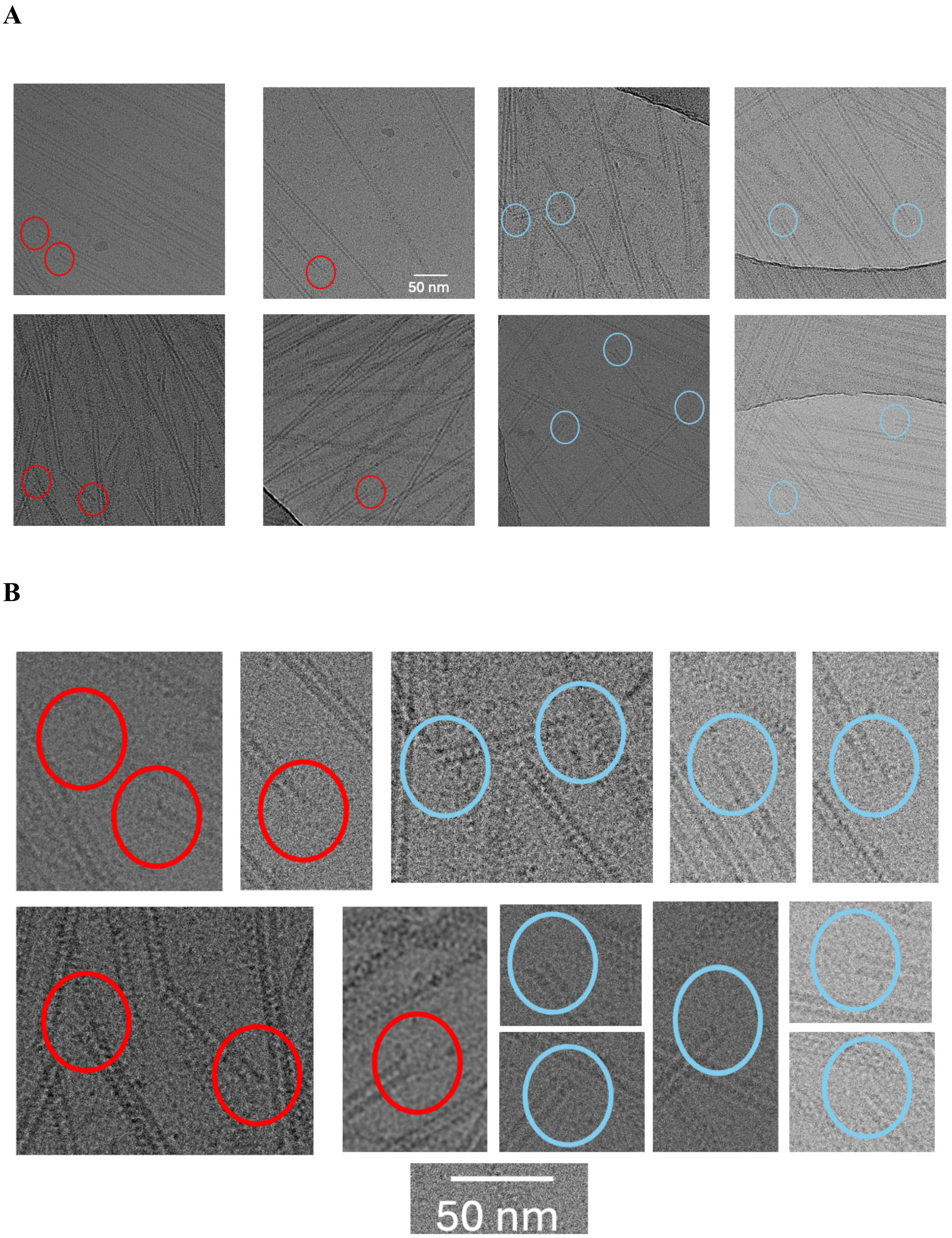

Fig. S6. Cryo-EM images of *Heim*-α/β-tubulin filament ends. (A) Some filament ends (red circles) exhibited frayed conformations with short, curved protofilaments extending away from the tubule. However, most filaments had blunt ends (blue circles). **(B)** Three-fold enlargement of the filament ends from **(A)**.

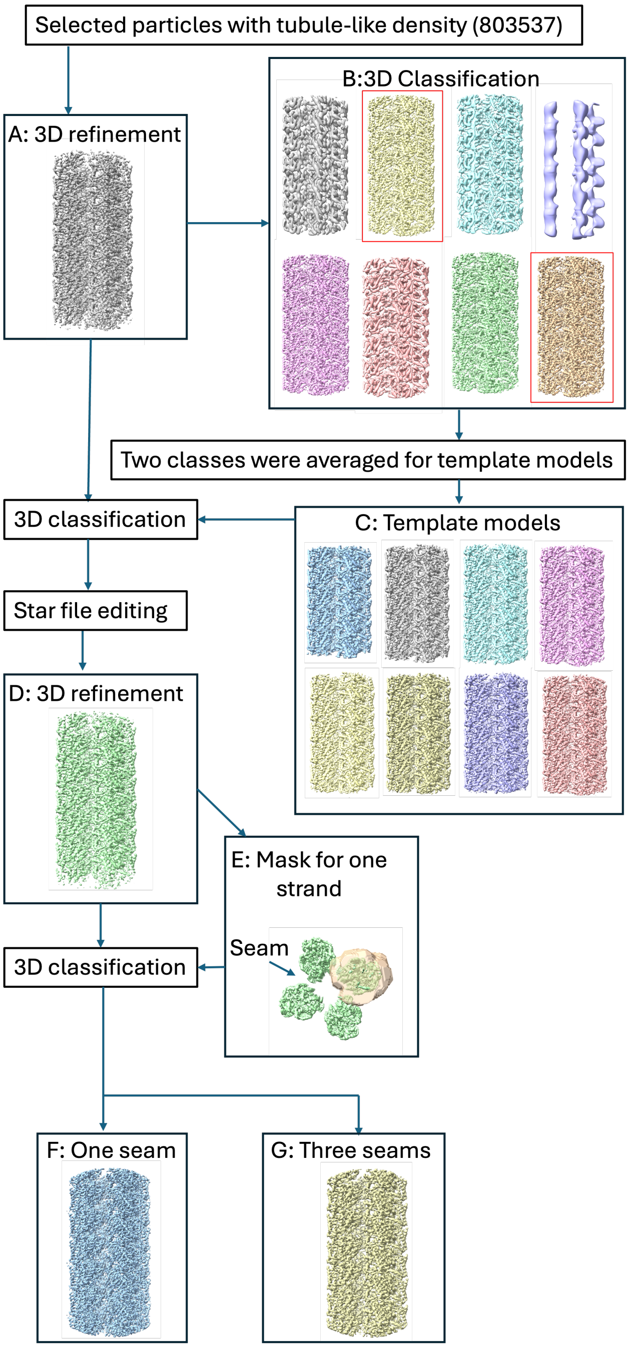

Fig. S7. Schematic diagram of *Heim*-α/β-microtubule image analysis

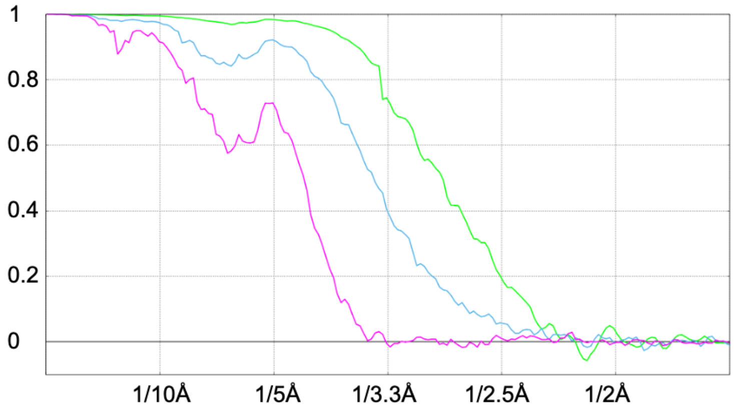

Fig. S8. Fourier shell correlation (FSC) curves using the 0.143 criterion. Green: averaged single-strand map derived from the one-seam reconstruction (2.4 Å). Cyan: whole filament map with one seam (2.8 Å). Magenta: whole filament map with three seams (3.9 Å).

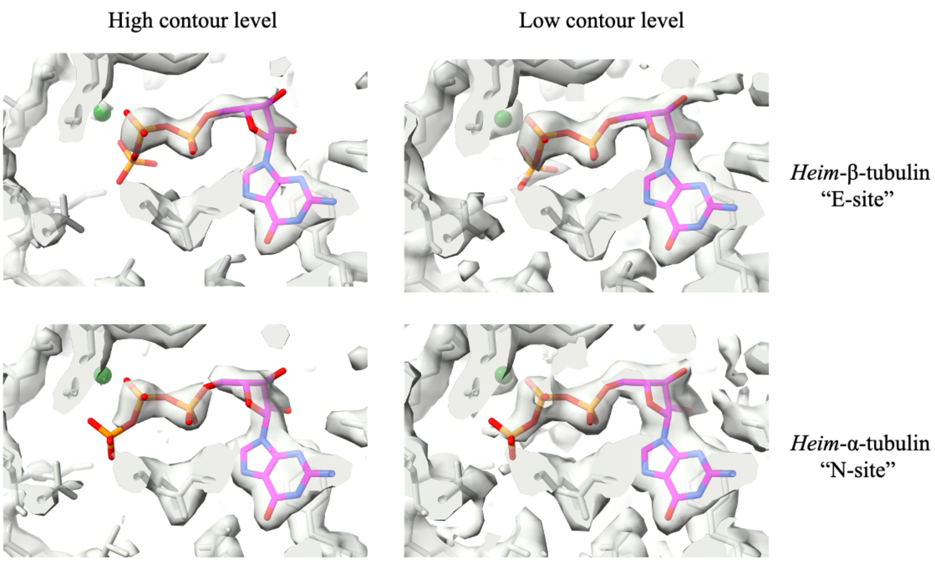

Fig. S9. Density at nucleotide-binding sites. *Heim*-α/β-tubulin did not show strong density for the γ-phosphate indicating GTP at either the *Heim*-α-tubulin “N-site” or *Heim*-β-tubulin “E-site”. At low contour levels, some evidence of the γ-phosphate was observed in *Heim*-α-tubulin “N-site” suggesting this site can accept GDP or GTP.

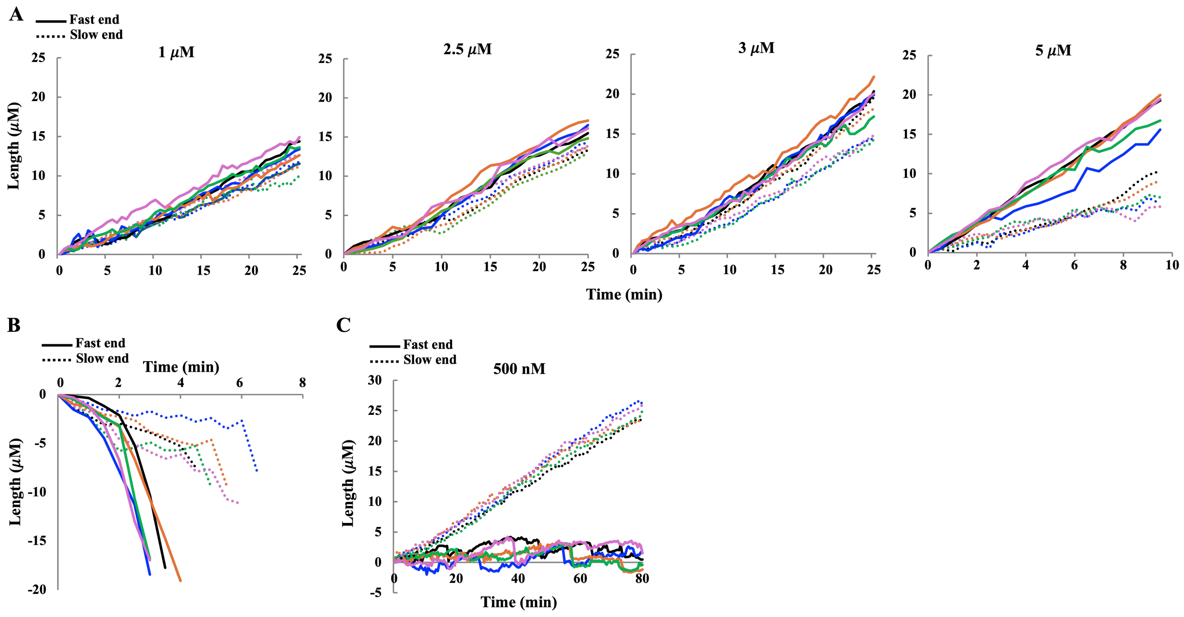

Fig. S10. Time courses of the fast and slow growing ends of of *Heim*-α/β-tubulin microtubules. (**A**) Polymerization at various concentrations of *Heim*-α/β-tubulin (1-5 μM). (**B**) Depolymerization observed after washing out free *Heim*-α/β-tubulin from the observation chamber. (**C**) Polymerization at 500 nM of *Heim*-α/β-tubulin showing dynamic instability properties. Lines with the same color correspond to the same filament. $n = 5$ filaments.

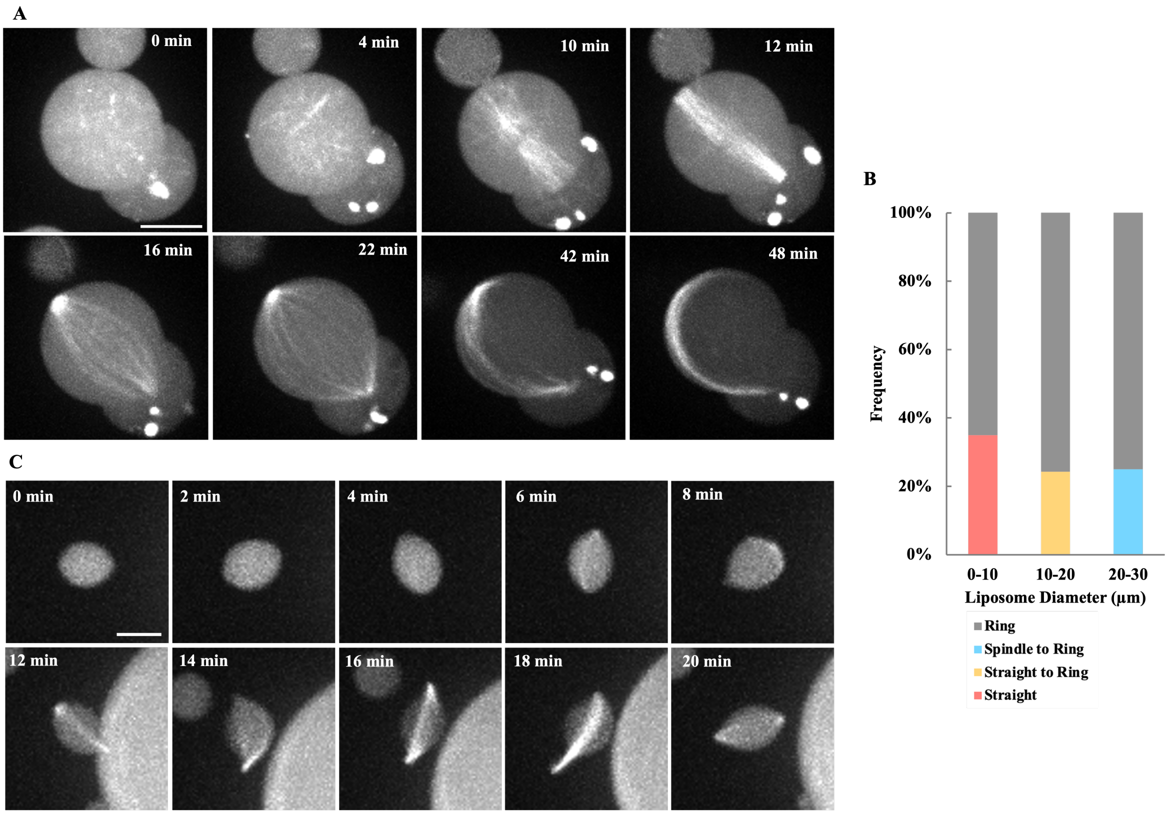

Fig. S11. Time-lapse images of *Heim*-α/β-tubulin-encapsulated liposomes. (**A**) Spindle-like structure was observed in a large liposome (~20 µm diameter). Scale bar, 10 µm. (**B**) Quantification of *Heim*-α/β-microtubule network structure inside liposomes along with morphological changes. (**C**) Membrane protrusion was formed by *Heim*-α/β-microtubule polymerization inside a small liposome (~5 µm diameter). Scale bar, 5 µm.

	#1 Heim-α/β-microtubule with single seam (EMDB-69786) (PDB 24RT)	#2 Averaged protofilament from #1 (EMDB-69801) (PDB 24SV)	#3 Heim-α/β-microtubule with three seams (EMDB-69802) (PDB 24SW)
**Data collection and processing**			
Voltage (kV)	300 kV	300 kV	300 kV
Electron exposure (e–/Å^2)	50	50	50
Defocus range (μm)	-0.3 ~ -4.0	-0.3 ~ -4.0	-0.3 ~ -4.0
Pixel size (Å)	0.75294	0.75294	0.75294
Phase plate	No	No	No
Symmetry imposed	Helical	Helical	Helical
Final particle images (no.)	223429	223429	71307
Map resolution (Å)	2.8	2.4	3.9
FSC threshold	0.143	0.143	0.143
Map resolution range (Å)	∞ ~ 2.8	∞ ~ 2.4	∞ ~ 3.9
**Refinement**			
Initial model used (PDB code)	24SV	AlphaFold3	24SV
Map sharpening *B* factor (Å^2)	-50	-76	-124
Model composition			
Non-hydrogen atoms	52520	13112	52520
Protein residues	6734	1682	6736
Ligands	32	8	32
R.m.s. deviations			
Bond lengths (Å)	0.005	0.005	0.005
Bond angles (°)	1.047	0.965	1.049
Validation			
MolProbity score	1.48	1.54	1.43
Clashscore	4.19	4.61	3.72
Poor rotamers (%)	0.83	1.13	0.75
Ramachandran plot			
Favored (%)	96	96.06	96.08
Allowed (%)	3.85	3.70	3.80
Disallowed (%)	0.15	0.24	0.12

Table S1. Cryo-EM data collection, processing and structure statistics.

Movie legends

Movie S1: Polymerization of *Heim*-α/β-microtubules observed by TIRF microscopy. Wide field view of the elongation of *Heim*-α/β-microtubules under the conditions outlined in Fig. 5A. Scale bar, 10 µm.

Movie S2: Depolymerization of *Heim*-α/β-microtubules observed by TIRF microscopy. Wide field view of the depolymerization of *Heim*-α/β-microtubules under the conditions outlined in Fig. 5B. Scale bar, 10 µm.

Movie S3. Dynamic instability of *Heim*-α/β-microtubules observed by TIRF microscopy. Wide field view of *Heim*-α/β-microtubules exhibiting dynamic instability under the conditions outlined in Fig. 5E. Scale bar, 10 µm.

Movie S4: Bundling of *Heim*-α/β-microtubules inside a large liposome observed by confocal microscopy. Polymerization of *Heim*-α/β-microtubules was induced inside liposomes under conditions outlined in Fig. 6. Scale bar, 10 µm.

Movie S5: Membrane protrusion to oval transition induced by *Heim*-α/β-microtubule polymerization inside a liposome observed by confocal microscopy. Polymerization of *Heim*-α/β-microtubules was induced inside liposomes under conditions outlined in Fig. 6. Scale bar, 10 µm

Movie S6. Formation of a membrane protrusion by *Heim*-α/β-microtubule polymerization inside a small liposome observed by confocal microscopy. Polymerization of *Heim*-α/β-microtubules was induced inside liposomes under conditions outlined in Fig. 6. Scale bar, 5 µm.

Movie S7. Formation of a spindle-like structure of *Heim*-α/β-microtubules inside a large liposome observed by confocal microscopy. Polymerization of *Heim*-α/β-microtubules was induced inside liposomes under conditions outlined in Fig. 6. Scale bar, 10 µm.
